# Hospital and health-center bed expansion and persistent relative mismatch across geometry-stable non-district Chinese counties, 2015–2023

**DOI:** 10.3389/fpubh.2026.1970301

**Published:** 2026-10-16

**Authors:** Kai Dai, Ziao Chen, Ling Wang, Zihao Lin, Yiqiang Feng

**Affiliations:** 1 School of Law, Central China Normal University, Wuhan, China; 2 School of Marxism, Sichuan Agricultural University, Chengdu, China; 3 Institute of Medical Information & Library, Chinese Academy of Medical Sciences and Peking Union Medical College, Beijing, China; 4 College of Law, Sichuan Agricultural University, Ya'an, China

**Keywords:** Chinese counties, hospital beds, local fiscal capacity, persistent relative mismatch, spatial mismatch, territorial health equity

## Abstract

**Introduction:**

Territorial health equity depends not only on expanding health resources but also on aligning local capacity with territorial planning contexts. We examined whether changes in hospital and health-center bed allocation were associated more strongly with territorial planning context or local fiscal-economic capacity.

**Methods:**

We analyzed a 2015-2023 panel of 1,787 geometry-stable non-district counties in China. The analysis combined county and province-by-year fixed effects, long-difference models, block-level Shapley decomposition, high-mismatch-state transitions, spatial diagnostics, discrete-time exit models, and adjacent-county pair comparisons.

**Results:**

Median beds per 10,000 registered residents increased from 35.1 in 2015 to 54.0 in 2023, whereas the distribution of relative planning-supply mismatch changed little. Planning-context and capacity blocks explained similarly small shares of annual variation in total beds. For bed density, the larger baseline planning-context share was driven primarily by the negative population coefficient and did not indicate allocation aligned with observed healthcare need. Higher initial capacity was associated with greater cumulative bed-density growth, lower relative mismatch, and a higher probability of exiting the high-mismatch state. Among county-years previously classified as high-mismatch, 84.0% remained in that state in the following year. Relative mismatch remained spatially clustered, but prefecture boundaries did not significantly amplify capacity-related bed-density gaps.

**Discussion:**

Aggregate expansion, relative territorial alignment, and recovery from persistent high mismatch represent distinct health-system outcomes. The findings support context-sensitive allocation, capacity-sensitive financing, and duration-sensitive monitoring, while remaining associational rather than causal.

## 1 Introduction

Equitable health systems are not defined by equal resource counts across places, but by whether territorial capacity aligns with population needs. This distinction separates horizontal equality, which concerns similar treatment for populations with comparable needs, from vertical equity, under which places facing greater needs may legitimately require more resources (1).

Spatial accessibility has evolved from simple provider-to-population ratios and nearest-distance measures toward multidimensional approaches that incorporate service supply, competing demand, transport impedance, topography, and facility hierarchy (2–4). Transport networks and topography can therefore mediate the relationship between a territorially measured planning context and effective access. In this study, the planning-context proxy should therefore be interpreted as a territorial allocation context rather than as a direct measure of realized healthcare demand.

From an accessibility perspective, the inverse care law suggests that health services may become concentrated in areas where populations are better placed to secure them rather than where health needs are greatest (5). From the perspective of health-system sustainability, such misalignment matters because persistent local high-mismatch conditions can weaken access, increase pressure on neighboring facilities, and undermine a health system's ability to provide consistently available services (6, 7). Hospital beds do not fully capture service quality, workforce availability, or actual utilization. Nevertheless, beds in hospitals and health centers remain a fundamental and consistently used input for planning. Their territorial distribution therefore provides a useful lens for examining whether health-system expansion is being translated into better-aligned local capacity.

From a systems perspective, health resource allocation results from interactions among demographic demand, public finance, local economic conditions, inherited infrastructure, administrative responsibilities, and spatial context, rather than from any single determinant (8). These interactions also unfold dynamically. Existing infrastructure constrains further expansion; local governments differ in their ability to finance and maintain additional capacity; and adjacent jurisdictions may face similar constraints because they share regional economic and institutional environments. Consequently, an increase in the national bed stock does not necessarily imply improved territorial alignment. A county may add beds while remaining in a relatively high-mismatch position compared with similar counties, whereas another may experience little absolute growth yet move out of the high-mismatch state. Assessing system performance therefore requires attention not only to bed levels but also to adjustment, mismatch transitions, and the persistence of relative disadvantage.

China provides an important setting for investigating these relationships. Successive health reforms have expanded insurance coverage, public financing, and the health service delivery network, yet substantial urban–rural, regional, and socioeconomic inequalities persist (8–10). National policy objectives are implemented through a multilevel administrative system in which subnational governments undertake substantial expenditure and service-delivery responsibilities. County governments operate within common national and provincial policy frameworks but differ considerably in fiscal resources, budgetary conditions, economic development, and household financial capacity. These differences may affect their ability to construct, maintain, and staff health facilities, even when territorial planning contexts are comparable.

Under China's hierarchical diagnosis and treatment system, a county's registered population may differ from the population seeking care and from the population represented in referral catchments (11–14). Large-scale population mobility may therefore allow effective capacity to remain concentrated in areas with stronger fiscal, institutional, and hospital-attraction capacity. This possibility makes county-level bed allocation a systems problem involving both the territorial planning context and local implementation capacity. Previous assessments of China's health reforms have therefore emphasized that progress toward health equity depends not only on national policy design but also on the incentives and implementation capacity of subnational governments (8–10). The relevant allocation question is thus not simply whether counties received more beds, but whether changes in local bed capacity were more closely associated with the territorial planning context or with localities' capacity to mobilize and sustain resources.

Existing county-level research establishes several key features of the distribution of health resources in China. National accessibility estimates show that the practical availability of hospital care is jointly shaped by facility supply, population distribution, and travel conditions (4, 14). County-level econometric evidence documents substantial spatial clustering in hospital-bed density and positive associations between bed availability and indicators such as government revenue and household savings (15). Research using the health-resource density index (HRDI), which simultaneously adjusts for population and geographic area, likewise identifies large and persistent disparities in the allocation of hospital beds (16). Spatiotemporal analyses further reveal geographically heterogeneous hotspots and relationships between bed supply and local socioeconomic or environmental conditions (17). Studies of county-level health resource allocation also indicate that economic status materially contributes to unequal resource distribution and that aggregate expansion can coexist with widening absolute gaps between richer and poorer counties (18, 19). Collectively, these studies demonstrate that bed supply is unequal, spatially clustered, and linked to local socioeconomic conditions. However, they do not directly determine whether observed changes in bed capacity are more systematically aligned with the multidimensional territorial planning context or with multidimensional local capacity.

The distinction between territorial planning context and capacity is also institutionally significant. China's decentralized public-finance structure assigns extensive expenditure responsibilities to subnational governments, while their ability to provide local public services depends on own-source revenue, economic resources, and intergovernmental transfers (20, 21). Vertical fiscal imbalance may affect both the level and composition of local public expenditure (21), and long-standing spatial inequality in education and health provision has been linked to uneven territorial economic and fiscal foundations (22). Nevertheless, studies of fiscal decentralization and population health in China have yielded mixed conclusions (23, 24).

International evidence suggests that decentralization can improve responsiveness when authority, fiscal resources, and accountability are aligned, but can reproduce territorial inequity when local implementation capacity varies (25, 26). Health-system resilience therefore depends not only on aggregate resource levels but also on local systems' ability to maintain and adapt service inputs in response to demographic, fiscal, and public-health shocks (6, 7). This makes it essential to distinguish local implementation capacity from the causal effect of fiscal decentralization. In this study, fiscal and economic indicators are treated as observed components of the resource environment in which county health systems operate. They are not interpreted as exogenous institutional treatments, nor are their estimated coefficients taken as causal effects of decentralization.

Three limitations of the existing evidence motivate our analysis. First, comparing individual regression coefficients is insufficient to determine whether bed allocation is more closely associated with the territorial planning context or with capacity, because both constructs are multidimensional and their components are correlated. A formal comparison requires planning-context-only, capacity-only, and joint specifications estimated using common samples, together with block-level tests and measures of relative explanatory contribution. Second, annual adjustment, cumulative growth, and movement into or out of the high-mismatch state represent distinct system processes. A covariate associated with annual bed additions may not predict long-term growth, and a county may remain in a high-mismatch position despite repeated additions if comparable counties expand more rapidly. Third, spatial dependence must be distinguished from substantive interpretations that cannot be observed directly. Similar outcomes in neighboring counties may reflect common regional environments or spatially correlated constraints; without patient-flow data, they cannot be interpreted as evidence that residents compensate for local high-mismatch conditions by using services in adjacent counties. Similarly, whether administrative boundaries condition associations between neighboring counties is an empirical question rather than an assumption.

We therefore pose three research questions. First, to what extent are annual changes and longer-term growth in county hospital and health-center bed capacity associated with the territorial planning context and local fiscal and economic capacity? Second, how often do counties enter, exit, or persist in a relatively high-mismatch state, and are these transitions systematically associated with the territorial planning context and with capacity? Third, how spatially clustered is the relative planning–supply mismatch, and do bed-supply differences between adjacent counties vary with capacity differences or with prefecture-boundary status? Together, these questions frame county bed allocation as a dynamic territorial system in which inputs, adjustment processes, high-mismatch states, and spatial relationships can be examined within a common analytical structure.

To address these questions, we construct a 2015–2023 panel of 1,787 geometry-stable non-district counties in China. We measure the primary outcome as the number of beds in hospitals and health centers, and we construct territorial planning-context and local-capacity blocks. The planning-context block captures population scale, geographical dispersion, and agricultural structure, while the capacity block captures fiscal resources per capita, the local revenue–expenditure relationship, economic output per capita, and household savings per capita. We compare planning-context-only, capacity-only, and joint fixed-effects models using common estimation samples, joint significance tests, leave-one-block-out changes in within-*R*^2^, and a two-block Shapley decomposition. We then construct a relative planning–supply mismatch measure from within-province-year rankings, translate it into annual high-mismatch states, and examine entry, exit, persistence, and the cumulative duration of high-mismatch spells. Finally, we combine global and local spatial diagnostics with spatial fixed-effects specifications and test whether adjacent-county capacity gaps are differently associated with bed-supply gaps across prefecture boundaries.

This study makes three contributions. Conceptually, it reframes county-level bed inequality as a system-alignment problem between territorial planning context and implementation capacity rather than as a collection of isolated socioeconomic correlates. Empirically, it provides a formal block-level comparison of planning context and capacity and connects annual adjustment to longer-term growth and high-mismatch-state transitions. Spatially, it distinguishes observed geographical dependence from unobserved patient movement and subjects administrative-boundary conditioning to direct empirical testing. The design is descriptive and associational. It does not identify causal effects of territorial planning context, fiscal capacity, or administrative boundaries, and spatial dependence is not interpreted as evidence of cross-county patient flows or service compensation.

## 2 Materials and methods

### 2.1 Study design, data sources, and analytical sample

#### 2.1.1 Study design and unit of analysis

We constructed an annual county-level panel covering 2015–2023 to examine whether changes in hospital and health-center bed capacity were more closely associated with the territorial planning context or with local fiscal and economic capacity. The unit of analysis was the non-district county-level administrative unit. Eligible units comprised counties, autonomous counties, banners, autonomous banners, county-level cities, and other county-level units outside municipal districts. Municipal districts were excluded because their populations, financing arrangements, and hospital catchments are embedded in metropolitan systems and are not directly comparable to those of non-district counties.

Throughout the analysis, *i* indexes counties, *p* indexes provinces, and *t* indexes years. Let $Y_{it}^{B}$ denote the stock of beds in hospitals and health centers, $Y_{it}^{D}$ denote beds per 10,000 registered residents, *P*_*it*_ denote the territorial planning-context proxy, *C*_*it*_ denote the local fiscal-economic capacity index, *M*_*ipt*_ denote the relative planning–supply mismatch, and *H*_*ipt*_ denote the high-mismatch-state indicator. The annual adjustment models used county- and province-by-year fixed effects. Long-difference models used province fixed effects measured at baseline, and spatial models used a common contiguity structure derived from the geometry-stable county frame. All analyses used aggregate administrative statistics and excluded individual health records.

#### 2.1.2 Data integration and sample construction

The panel was compiled from the county and city volumes of the China County Statistical Yearbook, the accompanying county database, county population files, and county fiscal-support records. The source fields included hospital and health-center beds, registered population, administrative area, gross domestic product (GDP), primary-industry value added, general budget revenue, general budget expenditure, and household savings. Administrative names and six-digit codes were harmonized before merging. Monetary values were converted to 2015 prices using a chained consumer-price index.

The 2023 administrative classification identified 1,850 non-district county-level units, corresponding to 16,650 potential county-year observations. We compared annual county codes and geometries and retained 1,787 counties with stable analytical geometry throughout 2015–2023. This geometry-stable frame contained up to 16,083 county-year observations. We retained missing observations as missing and did not impute them. Each regression therefore reports its model-specific complete-case sample, and the planning-context-only, capacity-only, and joint models were estimated on an identical common sample.

The final mismatch analysis contained 16,022 non-missing county-year observations, the common long-difference sample contained 1,764 counties, the baseline exit model contained 3,591 county-year observations, the spatial panel contained 1,620 counties and 14,580 county-year observations, and the adjacent-county analyses contained 36,551 pair-years. No revised analysis exceeded the theoretical maximum of 16,083 county-year observations.

#### 2.1.3 Spatial frame and ethics

Official annual county boundaries were used to assess administrative-code and geometric stability. For each county, we compared its polygon with the corresponding polygon in the preceding year. A county was classified as geometry-stable when its minimum year-to-year polygon intersection-over-union was at least 0.90, its maximum absolute year-to-year area change did not exceed 5%, and its maximum year-to-year centroid displacement did not exceed 10 km. Maps were drawn against the GS(2024)0650 standard national base map. Restricting spatial analyses to geometry-stable counties prevented administrative reorganization from being mechanically recorded as a change in bed supply, mismatch, or neighborhood structure.

The study used only aggregated administrative data and did not involve individual health records or identifiable personal information. Institutional review-board approval and informed consent were therefore not applicable.

### 2.2 Measurement of bed capacity, territorial planning context, and mismatch

#### 2.2.1 Hospital-bed outcomes

The primary resource measure was the reported stock of beds in hospitals and health centers. The source did not permit a consistent separation of hospital beds from health-center beds over the complete study period. We therefore did not describe the outcome as primary healthcare resources or as a multi-resource health-service index.

Bed density was calculated as follows:


$$\begin{array}{l} Y_{it}^{D}=\frac{Y_{it}^{B}}{Pop_{it}}\times 10,000 \end{array}$$

where $Y_{it}^{B}$ denotes beds in hospitals and health centers, and *Pop*_*it*_ denotes registered population in persons. Annual adjustment was assessed using both the natural logarithm of total beds and the natural logarithm of beds per 10,000 registered residents:


$$\begin{array}{l} {\overset{\sim }{Y}}_{it}^{B}=\ln\left(Y_{it}^{B}\right)\\ {\overset{\sim }{Y}}_{it}^{D}=\ln\left(Y_{it}^{D}\right) \end{array}$$

The total-bed outcome captures aggregate local capacity, whereas the density outcome captures capacity relative to the registered population. Bed counts do not capture staffing, occupancy, turnover, service quality, realized utilization, or health outcomes.

Positive continuous variables used in the baseline regression models were winsorized at the pooled 1st and 99th percentiles before logarithmic transformation. Untrimmed outcomes were retained for robustness analyses.

Logarithmic transformations were applied only to strictly positive fiscal observations. One county-year record reported negative real fiscal revenue per capita and a negative revenue-to-expenditure ratio: county code 530128 in 2022, with values of −406.309 and −0.0497, respectively. This record was treated as invalid and set to missing for the fiscal component and composite capacity index. It was not log-transformed, replaced with an arbitrary positive value, or winsorized to the positive domain.

#### 2.2.2 Annual standardization

The planning-context and capacity components were standardized separately for each year. For component k, the annual standard score was calculated as follows:


$$\begin{array}{l} Z_{kit}=\frac{X_{kit}-{\bar{X}}_{kt}}{s_{kt}} \end{array}$$

where *X*_*kit*_ denotes the value of component *k* for county *i* in year *t*, ${\bar{X}}_{kt}$ denotes the national county mean, and *s*_*kt*_ denotes the corresponding standard deviation. The standardized components were subsequently combined into the territorial planning-context and local-capacity indices.

#### 2.2.3 Territorial planning-context block

The territorial planning-context proxy comprised population scale, geographic dispersion, and agricultural structure. Population scale, $P_{it}^{pop}$, was the annual standard score of log registered population. Geographic dispersion, $P_{it}^{disp}$, was the negative annual standard score of log population density, such that higher values represented greater spatial dispersion of the population. Agricultural structure, $P_{it}^{agr}$, was the annual standard score of the primary-industry share of GDP.


$$\begin{array}{l} P_{it}=\frac{P_{it}^{pop}+P_{it}^{disp}+P_{it}^{agr}}{3} \end{array}$$

The proxy represents a planning-oriented territorial construct rather than a direct measure of observed healthcare need. Its components capture demographic scale, settlement dispersion, and rural production structure. The agricultural-share component was treated as a continuous indicator of rural production and service-delivery context. It is not an official agricultural-county classification and does not directly measure morbidity, mortality, aging, or disease burden.

#### 2.2.4 Local-capacity block

Local capacity combined four annual standard scores: log real fiscal revenue per capita, $C_{it}^{fis}$; the fiscal revenue–expenditure ratio, $C_{it}^{aut}$; log real GDP per capita, $C_{it}^{gdp}$; and log real household savings per capita, $C_{it}^{sav}$. The equal-weight capacity index was calculated as follows:


$$\begin{array}{l} C_{it}=\frac{C_{it}^{fis}+C_{it}^{aut}+C_{it}^{gdp}+C_{it}^{sav}}{4} \end{array}$$

The capacity index describes the local fiscal-economic environment in which county health systems operate. It is not interpreted as an exogenous decentralization treatment, and its coefficients are not interpreted as causal effects of fiscal decentralization.

#### 2.2.5 Relative planning–supply mismatch

Relative planning–supply mismatch was constructed by comparing each county's territorial planning-context position with its bed-density position within the same province and year. For variable *X*, the province-by-year percentile rank was calculated as follows:


$$\begin{array}{l} R_{ipt}^{X}=100\times \frac{rank\left(X_{ipt}\right)-1}{n_{pt}-1} \end{array}$$

where *n*_*pt*_ denotes the number of counties with non-missing observations for variable *X* in province *p* and year *t*. Tied observations were assigned their average rank. When *X*=P, the rank represents the county's relative territorial planning-context position; when *X*=Y^D^, it represents the county's relative bed-density position.

The two percentile ranks were constructed within the same province and year and therefore placed territorial planning context and bed density on a common 0–100 scale. The relative mismatch measure was calculated as follows:


$$\begin{array}{l} M_{ipt}=R_{ipt}^{P}-R_{ipt}^{D} \end{array}$$

Positive values indicate that a county's territorial planning-context rank exceeded its bed-density rank, indicating a relatively less favorable planning–supply position within the same province and year. This measure represents a relative comparison and does not establish an absolute or clinically verified bed shortage.

#### 2.2.6 High-mismatch states and transitions

An annual high-mismatch state was assigned to observations in the highest quartile of the relative mismatch distribution:


$$\begin{array}{l} H_{it}=𝟙\left[M_{ipt}\geq q_{0.75,t}\right] \end{array}$$

where 1(·) is the indicator function and *q*_0.75, *t*_ is the annual 75th percentile of relative mismatch. The upper-quartile threshold is a distributional monitoring rule rather than a clinical or statutory bed standard. It identifies relative upper-tail membership and does not define an absolute or clinically verified bed shortage. Approximately one-quarter of valid observations was classified as high mismatch by construction.

One-year transitions were represented as follows:


$$\begin{array}{l} T_{ipt}=\left(H_{ip,t-1},H_{ipt}\right) \end{array}$$

The four transition categories were remaining outside the high-mismatch state, entering the high-mismatch state, exiting the high-mismatch state, and remaining in the high-mismatch state.

Consecutive high-mismatch spell duration was defined as follows:


$$\begin{array}{l} D_{ipt}=\overset{t-1}{\underset{s=0}{\sum }}1\left(H_{ip,t-s}=1\right) \end{array}$$

Study-period persistence was defined as follows:


$$\begin{array}{l} R_{i}=1\left(\underset{t}{\sum }H_{ipt}\geq 3\right) \end{array}$$

These indicators describe relative distributional persistence and do not represent verified persistent absolute shortage.

### 2.3 Territorial planning context vs. capacity in annual and cumulative adjustment

#### 2.3.1 Annual fixed-effects models

Annual adjustment models used 1-year-lagged territorial planning-context and capacity components. County fixed effects accounted for time-invariant local characteristics, and province-by-year fixed effects accounted for province-specific annual policies, macroeconomic conditions, fiscal shocks, and common reporting changes. The joint model was as follows:


$$\begin{array}{l} Y_{it}=\alpha_{i}+\gamma_{pt}+\beta_{P}^{\prime }P_{i,t-1}+\beta_{C}^{\prime }C_{i,t-1}+\varepsilon_{it} \end{array}$$

where *Y*_*it*_ denotes either log total beds or log beds per 10,000 registered residents, α_*i*_ denotes county fixed effects, γ_*pt*_ denotes province-by-year fixed effects, and *P*_*i, t*−1_ and *C*_*i, t*−1_ denote vectors of lagged planning-context and capacity components.

Separate planning-context-only, capacity-only, and joint models were estimated using the same complete-case sample. Standard errors were clustered by county. Joint block tests were reported because individual component coefficients do not, by themselves, establish whether bed adjustment is more closely associated with planning context or capacity.

Population scale and settlement dispersion exhibit limited within-county variation. Their fixed-effects coefficients are therefore identified from relatively small year-to-year deviations and may be sensitive to measurement error, short-run reporting variation, and regression-to-the-mean effects. These coefficients are interpreted as conditional longitudinal associations rather than stable structural effects.

#### 2.3.2 Block-level contribution

For each block *g*, the leave-one-block-out contribution was measured by the reduction in within-*R*^2^ when that block was removed from the joint model:


$$\begin{array}{l} \Delta R_{-g}^{2}=R_{\text{joint}}^{2}-R_{-g}^{2} \end{array}$$

Because the two blocks are correlated, we also calculated an exact two-block Shapley allocation. For block *g*, the allocation was calculated as the average of its marginal contribution across the two possible entry orders (27):


$$\begin{array}{l} \phi_{g}=\frac{1}{2}\left[\left(R_{g}^{2}-R_{\emptyset }^{2}\right)+\left(R_{\text{joint}}^{2}-R_{-g}^{2}\right)\right] \end{array}$$

where $R_{\emptyset }^{2}$ denotes the within-*R*^2^ of the model containing only fixed effects, $R_{g}^{2}$ denotes the within-*R*^2^ of the model containing only block *g*, $R_{\text{joint}}^{2}$ denotes the within-*R*^2^ of the joint model, and $R_{-g}^{2}$ denotes the within-*R*^2^ of the model excluding block *g*.

Shapley shares were calculated as follows:


$$\begin{array}{l} Share_{g}=\frac{\phi_{g}}{\sum_{h}\phi_{h}} \end{array}$$

The Shapley decomposition was treated as exploratory model-fit accounting. It is sensitive to the included components, outcome definition, population denominator, and model specification and is not interpreted as definitive evidence of substantive importance.

#### 2.3.3 Long-difference models

Long-difference models examined whether initial territorial planning context and capacity were associated with cumulative change between 2015 and 2023:


$$\begin{array}{l} \Delta Y_{i}=\alpha +\delta_{P}P_{i,2015}+\delta_{C}C_{i,2015}+\lambda Y_{i,2015}+\eta_{p\left(i\right)}+\varepsilon_{i} \end{array}$$

where Δ*Y*_*i*_ = *Y*_*i*, 2023_ − *Y*_*i*, 2015_, *Y*_*i*, 2015_ denotes the outcome-specific baseline control and η_*p*(*i*)_ denotes 2015 province fixed effects. Separate models were estimated for changes in log total beds, log beds per 10,000 residents and relative planning–supply mismatch. The long-difference estimand captures cumulative adjustment from initial conditions and is therefore distinct from short-run within-county adjustment.

### 2.4 High-mismatch-state persistence and exit

#### 2.4.1 Observed transition matrix

We first tabulated the complete 1-year transition matrix defined above. Counts and row percentages were reported for counties remaining outside the high-mismatch state, entering the high-mismatch state, exiting the high-mismatch state, and remaining in the high-mismatch state. Row percentages were conditioned on the previous year's state and therefore provide directly interpretable entry and exit rates. Missing mismatch observations were retained as missing; transitions were calculated only when both adjacent-year states were observed.

#### 2.4.2 Annual high-mismatch-state model

The annual probability of occupying the high-mismatch state was estimated using a fixed-effects linear-probability model:


$$\begin{array}{l} H_{ipt}=\alpha_{i}+\gamma_{pt}+\beta_{P}P_{i,t-1}+\beta_{C}C_{i,t-1}+\varepsilon_{ipt} \end{array}$$

The model included county fixed effects and province-by-year fixed effects, with standard errors clustered by county. It estimated average within-county associations between changes in the explanatory variables and high-mismatch status rather than a structural or causal probability mechanism.

#### 2.4.3 Conditional high-mismatch-state exit model

Exit models were estimated only for county-years in which the countries had been in the high-mismatch state in the preceding year. The baseline logistic specification was as follows:


$$\begin{array}{l} \mathrm{Pr}\left(E_{ipt}=1\right)={\text{logit}}^{-1}(\alpha +\beta_{P}P_{i,t-1}+\beta_{C}C_{i,t-1}\\ \;+\beta_{D}D_{ip,t-1}+\gamma_{t}+\eta_{p}+\varepsilon_{ipt}) \end{array}$$

where *E*_*ipt*_ = 1 indicates exit from the high-mismatch state, *D*_*ip, t*−1_ denotes prior consecutive high-mismatch spell duration, γ_*t*_ denotes year fixed effects, and η_*p*_ denotes province fixed effects. Standard errors were clustered at the county level.

To examine whether the capacity association differed by agricultural structure, we defined the following:


$$\begin{array}{l} A_{i}=𝟙\left(P_{i,2015}^{agr}\geq q_{0.67}\right) \end{array}$$

where *A*_*i*_ = 1 identifies counties in the highest tercile of the 2015 primary-industry share. The interaction *C*_*i, t*−1_ × *A*_*i*_ tests whether the association between capacity and exit differs according to baseline agricultural structure. Average adjusted exit probabilities were calculated at capacity values of −1, 0, and +1 standard deviations and at prior high-mismatch spell durations of 1, 3, and 5 years.

Because counties observed in longer high-mismatch spells are selected survivors that have not exited in previous periods, the duration coefficient is interpreted as a conditional association rather than evidence of a causal cumulative disadvantage.

### 2.5 Spatial dependence and prefecture-boundary conditions

#### 2.5.1 Spatial weights and exploratory diagnostics

A Queen-contiguity matrix was constructed from the 2023 geometry of counties that remained stable throughout the study period. Two counties were treated as neighbors if their polygons shared an edge or a vertex. The matrix was row-standardized. Eleven island counties without a Queen neighbor were retained in all non-spatial analyses but omitted from analyses requiring a connected spatial lag.

Annual global spatial autocorrelation in relative planning–supply mismatch was measured using Moran's I (28):


$$\begin{array}{l} I_{t}=\frac{n_{t}}{S_{0}}\frac{\sum_{i}\sum_{j}w_{ij}\left(M_{it}-{\bar{M}}_{t}\right)\left(M_{jt}-{\bar{M}}_{t}\right)}{\sum_{i}{\left(M_{it}-{\bar{M}}_{t}\right)}^{2}} \end{array}$$

where *M*_*it*_ denotes relative planning–supply mismatch rather than absolute shortage, *n*_*t*_ denotes the number of counties with non-missing mismatch values, *w*_*ij*_ denotes the row-standardized Queen-contiguity weight, and *S*_0_ denotes the sum of all spatial weights. Statistical significance was evaluated using 999 random permutations.

Local Moran statistics were calculated for the 2015 and 2023 endpoints (29):


$$\begin{array}{l} I_{it}^{L}=\left(M_{it}-{\bar{M}}_{t}\right)\underset{j}{\sum }w_{ij}\left(M_{0jt}-{\bar{M}}_{t}\right) \end{array}$$

Counties with local permutation *p*-values below 0.05 were classified as high–high relative mismatch clusters, low–low relative mismatch clusters, high–low spatial outliers, or low–high spatial outliers. These classifications describe local spatial association and do not identify causal mechanisms, patient flows, or cross-county service compensation.

#### 2.5.2 Spatial panel model

Conditional spatial dependence in bed density was examined using a spatial-lag panel model among counties with complete 9-year data and at least one Queen-contiguity neighbor:


$$\begin{array}{l} Y_{it}^{D}=\rho \underset{j}{\sum }w_{ij}Y_{jt}^{D}+\beta_{P}P_{it}+\beta_{C}C_{it}+\alpha_{i}+\gamma_{pt}+\varepsilon_{it} \end{array}$$

where $Y_{it}^{D}$ denotes log beds per 10,000 registered residents, ρ denotes the spatial-lag association, *P*_*it*_ and *C*_*it*_ denote the planning-context and capacity indices, α_*i*_ denotes county fixed effects, and γ_*pt*_ denotes province-by-year fixed effects.

The preferred specification included county fixed effects and province-by-year fixed effects. The province-by-year effects accounted for province-specific annual policies, macroeconomic conditions, fiscal shocks, and common reporting changes. A less-saturated sensitivity specification included county fixed effects and national-year fixed effects.

The preferred model produced ρ = 0.0903, whereas the specification with national-year effects produced ρ = 0.2140. An earlier specification without temporal effects was not retained because it did not control for common temporal trends.

The spatial-lag coefficient is interpreted as the residual spatial association after conditioning on observed planning context, local capacity, and temporal effects. It is not interpreted as a causal spillover, cross-county patient flow, or service compensation.

#### 2.5.3 Adjacent-county pair models

For every adjacent-county pair *i* and *j*, we calculated annual differences in log bed density, relative planning–supply mismatch, territorial planning context, and local fiscal-economic capacity. We also identified whether the two counties belonged to different prefecture-level jurisdictions.

The absolute gap for variable *X* was defined as follows:


$$\begin{array}{l} G_{ijt}^{X}=|X_{it}-X_{jt}| \end{array}$$

The absolute bed-density-gap model was specified as follows:


$$\begin{array}{l} G_{ijt}^{Y}=\theta_{P}G_{ijt}^{P}+\theta_{C}G_{ijt}^{C}+\theta_{B}\left(G_{ijt}^{C}\times B_{ij}\right)+\mu_{ij}+\tau_{t}+e_{ijt} \end{array}$$

where $G_{ijt}^{Y}$ denotes the absolute gap in log beds per 10,000 residents, $G_{ijt}^{P}$ denotes the territorial planning-context gap, $G_{ijt}^{C}$ denotes the capacity gap, *B*_*ij*_ = 1 if the pair crosses a prefecture-level boundary and *B*_*ij*_ = 0 otherwise, μ_*ij*_ denotes pair fixed effects and τ_*t*_ denotes year fixed effects. An analogous specification was estimated using relative planning–supply mismatch as the dependent variable.

Because the same county may appear in multiple adjacent pairs, dyadic observations sharing either endpoint are not independent. Standard errors were therefore multiway clustered on both county endpoints rather than clustered only at the pair level. Because cross-prefecture status is time invariant, its main effect is absorbed by pair fixed effects. The interaction coefficient tests whether the capacity-gap association differs across prefecture boundaries. This is a border-conditioned association test and not a geographic regression-discontinuity design.

#### 2.5.4 Directional adjacent-county comparisons

Absolute gaps indicate the magnitude of disparity but not which county has the relative advantage. We therefore estimated a directional specification in which each adjacent pair was oriented from the lower-capacity county to the higher-capacity county. Let *h* denote the higher-capacity endpoint and *l* denote the lower-capacity endpoint within pair *i, j* and year *t*.

The directional capacity advantage and bed-density advantage were defined as follows:


$$\begin{array}{l} A_{ijt}^{C}=C_{h,t}-C_{l,t}\\ A_{ijt}^{D}=Y_{h,t}^{D}-Y_{l,t}^{D} \end{array}$$

The directional model was specified as follows:


$$\begin{array}{l} A_{ijt}^{D}=\theta_{C}A_{ijt}^{C}+\theta_{B}\left(A_{ijt}^{C}\times B_{ij}\right)+\mu_{ij}+\tau_{t}+e_{ijt} \end{array}$$

A positive θ_*C*_ indicates that the higher-capacity county tends to have higher bed density. The interaction coefficient tests whether this directional association differs across prefecture boundaries. Standard errors were multiway clustered on both county endpoints.

### 2.6 Robustness, diagnostics, and statistical implementation

#### 2.6.1 Alternative high-mismatch and relative mismatch definitions

Robustness analyses varied the annual high-mismatch threshold from the highest 25% of the relative mismatch distribution to the highest 20% and 30%. These thresholds represent distributional monitoring rules rather than clinical or statutory standards.

Alternative planning-context indices assigned 50% weight to population scale or settlement dispersion, while assigning 25% to each remaining component. To address the mechanical overlap between population in the planning proxy and population in the bed-density denominator, we constructed a no-population planning-context proxy:


$$\begin{array}{l} P_{it}^{NP}=\frac{P_{it}^{disp}+P_{it}^{agr}}{2} \end{array}$$

$P_{it}^{NP}$ excludes population scale and is used to test whether the baseline planning–supply mismatch is mechanically driven by the presence of population in both the planning proxy and the bed-density denominator. It is not an alternative healthcare-need index.

For the no-population specification, the relative mismatch was defined as follows:


$$\begin{array}{l} M_{ipt}^{NP}=R_{ipt}^{P^{NP}}-R_{ipt}^{D} \end{array}$$

where $R_{ipt}^{P^{NP}}$ denotes the province-by-year rank of the no-population planning-context proxy. The corresponding high-mismatch state, transition indicators, spell duration, exit outcomes and spatial diagnostics were reconstructed using $M_{ipt}^{NP}$.

We also ranked supply using the Health Resource Density Index, calculated as beds divided by the square root of the product of registered population in units of 10,000 and county area in square kilometers (16):


$$\begin{array}{l} HRDI_{it}=\frac{Y_{it}^{B}}{\sqrt{Pop_{it}/10,000\times Area_{i}}} \end{array}$$

A model-based alternative defined relative mismatch as the negative residual from a province-by-year demeaned regression of log bed density on the planning-context components. Correlations and high-mismatch-state agreement with the baseline definition were reported for every alternative construction.

#### 2.6.2 Outcome, sample, and component diagnostics

Outcome and sample checks used untrimmed bed density, excluded counties with fewer than 20,000 registered residents, removed the upper and lower 1% of bed-density observations, and used the HRDI-adjusted bed supply as an alternative primary outcome. An expanded planning-context proxy additionally incorporated income disadvantage for counties where rural-income data were available; this variable was not used to define the national baseline sample.

Component diagnostics reported pooled correlations, variance inflation factors, and within- and between-county standard deviations. These diagnostics were used to limit component-level interpretation and to motivate the emphasis on block-level associations rather than individual coefficient rankings.

Because registered population contributes to both the baseline planning-context proxy and the denominator of bed density, the population-exclusion analysis was treated as a central construct-sensitivity test. The no-population mismatch was correlated with the baseline mismatch at 0.870, and agreement in high-mismatch-state classification was 86.9%. The principal component analysis (PCA) and Mundlak specifications described below were used as complementary diagnostics rather than as replacements for the prespecified equal-weight indices.

#### 2.6.3 Complementary association and shock analyses

Pooled principal component analyses were used to assess sensitivity to equal weighting. PCA scores were constructed separately for the planning-context and capacity components after standardization. The component signs were oriented for interpretability, and the scores were not optimized using the bed outcomes. The planning PC1 explained 63.5% of the component variance, whereas the capacity PC1 explained 76.2%. The planning PC1 correlated 0.298 with the equal-weight planning-context proxy, while the capacity PC1 correlated 0.9998 with the equal-weight capacity index.

To distinguish longitudinal associations from persistent cross-county differences, correlated-random-effects/Mundlak models decomposed each explanatory variable as follows:


$$\begin{array}{l} X_{it}=\left(X_{it}-{\bar{X}}_{i}\right)+{\bar{X}}_{i} \end{array}$$

The generic Mundlak specification was as follows:


$$\begin{array}{l} Y_{it}=\beta_{W}\left(X_{it}-{\bar{X}}_{i}\right)+\beta_{B}{\bar{X}}_{i}+\alpha_{i}+\gamma_{t}+\varepsilon_{it} \end{array}$$

where $X_{it}-{\bar{X}}_{i}$ represents the within-county deviation, and ${\bar{X}}_{i}$ represents the between-county mean. β_*W*_ captures the within-county association, and β_*B*_ captures the persistent between-county association. These estimates were interpreted as complementary descriptive evidence because the within-county variation of several planning-context components was limited.

Pandemic-period heterogeneity was examined by interacting lagged planning-context and capacity indices with indicators for 2016–2019, 2020–2022, and 2023. The 2016–2019 period was used as the pre-pandemic reference period. Formal interaction contrasts tested whether the 2020–2022 and 2023 associations differed from those in the pre-pandemic period.

As a complementary duration analysis, we estimated a discrete-time complementary log–log model among county-years in which the county occupied the high-mismatch state in the preceding year:


$$\begin{array}{l} \log\left[-\log\left(1-h_{ipt}\right)\right]=\alpha +\beta_{C}C_{i,t-1}+\overset{5+}{\underset{d=2}{\sum }}\lambda_{d}D_{i,t-1}^{d}+\gamma_{t}+\eta_{p} \end{array}$$

where *h*_*ipt*_ denotes the conditional hazard of exiting the high-mismatch state, *C*_*i, t*−1_ denotes lagged capacity, $D_{i,t-1}^{d}$ denotes categorical high-mismatch spell duration, and the 1-year spell category serves as the reference group. This specification allows the baseline exit hazard to vary flexibly across spell lengths. Because counties with longer spells are selected survivors that have not exited in previous periods, duration coefficients are interpreted as conditional hazard differences rather than evidence of causal duration dependence.

#### 2.6.4 Statistical reporting and software

All tests were two-sided. We reported coefficients, standard errors, confidence intervals, exact *p*-values where practicable, sample sizes, and model-fit statistics. Results with *p* < 0.10 were treated as suggestive rather than statistically significant.

Python 3.8.19 (Python Software Foundation, Wilmington, DE, USA) was used for data integration, construction of spatial weights, global and local spatial statistics, PCA diagnostics, population-exclusion spatial analyses, and all final figures. Fixed-effects, long-difference, transition, exit, Mundlak, discrete-time survival, and adjacent-county models were estimated in Stata 17 MP.

The study is descriptive and associational. No coefficient is interpreted as the causal effect of territorial planning context, local capacity, spatial dependence, administrative boundaries, or high-mismatch duration. The high-mismatch state is a relative distributional classification and is not interpreted as an absolute or clinically verified bed shortage.

## 3 Results

### 3.1 Analytical coverage and changes in hospital and health-center bed supply

Following the variable definitions in Table 1, Panel A, the geometry-stable analytical sample comprised 1,787 non-district counties observed from 2015 to 2023, corresponding to a theoretical maximum of 16,083 county-year observations. Core-variable completeness ranged from 98.8% for the capacity index to 99.7% for beds and registered population. A relative planning–supply mismatch was observed in 16,022 county-years. The analytical frame retained broad territorial coverage while excluding municipal districts and changes arising from substantial county-boundary reorganization (Figure 1). Descriptive statistics for the geometry-stable county-year sample are reported in Table 1, Panel B.

Table 1Variable definitions, descriptive statistics, and data sources.

**Table:** 

Panel A. Variable definitions and data sources			
Construct	Variable	Definition	Source
Hospital and health-center beds	beds	Beds in hospitals and health centers	County yearbook database
Beds per 10,000	beds_10k	Hospital and health-center beds/registered population × 10,000	Constructed
Planning context: population	need_population	Year-standardized log registered population	Constructed
Planning context: dispersion	need_dispersion	Negative year-standardized log population density	Constructed
Planning context: agriculture	need_agriculture	Year-standardized primary-industry share; continuous rural production/service-context indicator	Constructed
Planning-context index	need_index3	Equal-weight mean of three territorial planning-context components	Constructed
Capacity: fiscal	cap_fiscal	Year-standardized log real fiscal revenue per capita; positive observations only	Constructed
Capacity: autonomy	cap_autonomy	Revenue/expenditure	Constructed
Capacity: economy	cap_gdp	Year-standardized log real GDP per capita	Constructed
Capacity: savings	cap_savings	Year-standardized log household savings per capita	Constructed
Capacity index	capacity_index	Equal-weight mean of four local-capacity components	Constructed
Relative planning–supply mismatch	mismatch	Within-province-year planning-context rank minus bed-density rank	Constructed
High-mismatch state	shortage25	Indicator for the annual upper quartile of relative planning–supply mismatch	Constructed
Population-weighted mismatch	mismatch_pop50	Mismatch using 50% population, 25% dispersion, and 25% agriculture planning-context weights	Robustness
Dispersion-weighted mismatch	mismatch_geo50	Mismatch using 25% population, 50% dispersion, and 25% agriculture planning-context weights	Robustness
HRDI mismatch	mismatch_hrdi	Planning-context rank minus HRDI-adjusted bed-supply rank	Robustness
Residual mismatch	mismatch_resid	Negative residual from the province-by-year-demeaned planning-context model of log bed density	Robustness

**Table:** 

Panel B. Descriptive statistics for the geometry-stable county-year sample						
Variable	*N*	Mean	SD	Median	Min.	Max.
Hospital beds	16,048	2,132	1,743	1,646	20.000	12,651
Beds per 10,000	16,028	46.796	20.263	43.827	0.842	500.000
HRDI-adjusted beds	16,028	6.803	4.820	5.908	0.035	92.222
Planning-context index	16,046	0.002	0.423	−0.026	−1.531	1.922
Capacity index	15,915	−0.011	0.871	−0.143	−2.263	3.441
Real fiscal revenue per capita	16,046	2,782	6,144	1,615	64.277	325,174
Fiscal autonomy	16,059	0.273	0.210	0.215	0.0057	2.992
Real GDP per capita	16,058	42,632	72,758	30,602	4,858	3,759,612
Real savings per capita	15,935	32,669	22,639	27,205	484.367	282,053
Agricultural share	16,069	0.200	0.114	0.185	0.006	0.755
Population density	16,052	289.569	288.240	189.922	0.112	3,371
Relative planning–supply mismatch	16,022	0.006	0.444	0.026	−0.990	0.992
High-mismatch state	16,022	0.253	0.435	0.000	0.000	1.000

Panel A: Territorial planning context is a planning-oriented contextual construct rather than observed morbidity-based healthcare need. The agricultural-share component is a continuous rural production and service-context indicator; no official agricultural-county classification is used. Hospital and health-center beds are the primary supply measure. Fiscal variables were log-transformed only for strictly positive observations. One non-positive fiscal county-year observation (county 530128, 2022; real fiscal revenue per capita = −406.309; fiscal autonomy = −0.0497) was set to missing for the fiscal component and composite capacity index. HRDI denotes the population-and-area-adjusted health-resource density index.

Panel B: Statistics use all non-missing observations in the geometry-stable sample. Monetary variables are expressed in 2015 prices. The high-mismatch state equals one for the annual upper quartile of relative planning–supply mismatch and is not an absolute or clinically verified bed-shortage standard. After excluding the non-positive fiscal record, the minimum positive fiscal revenue per capita is 64.277, and the minimum positive fiscal-autonomy ratio is 0.0057.

**Figure 1:**
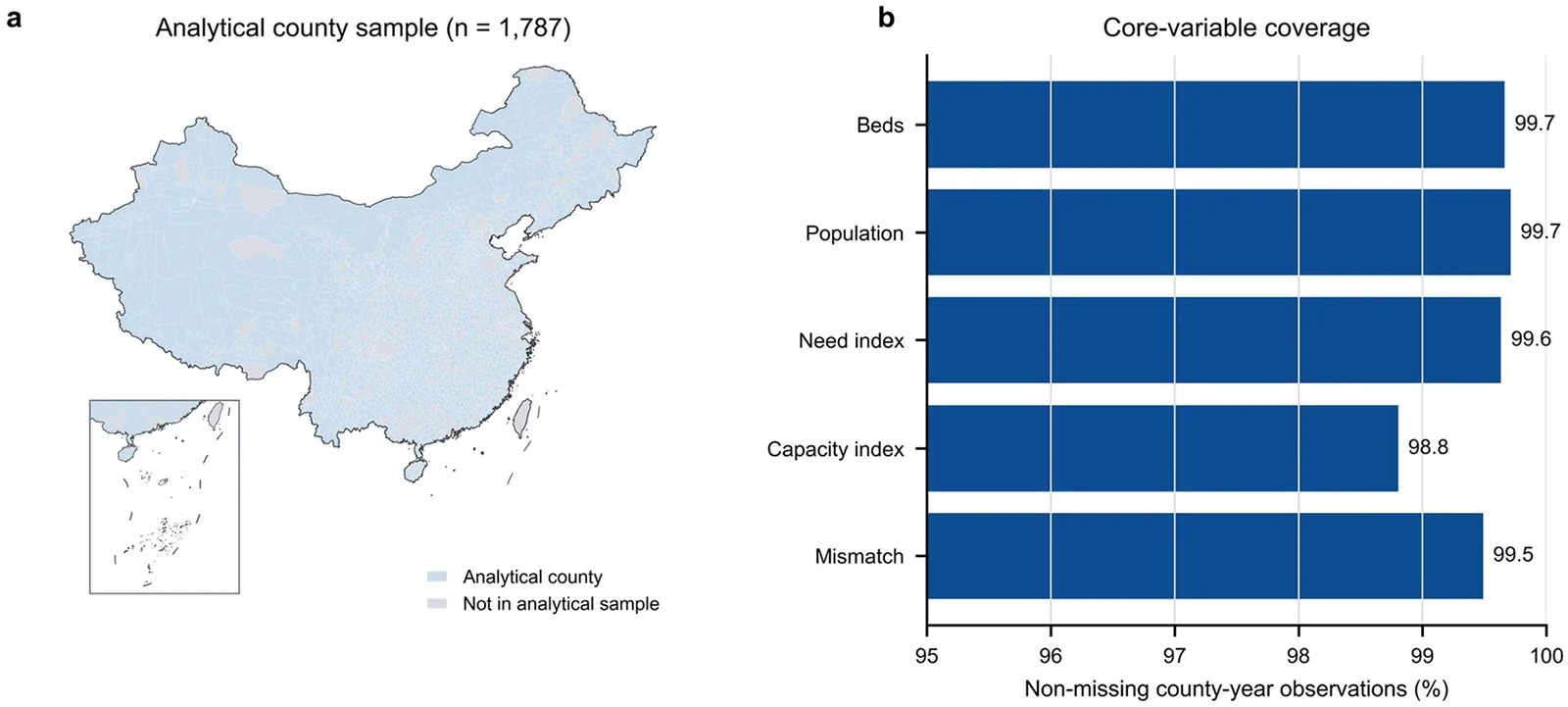
Geometry-stable analytical sample and data coverage. **(a)** The 1,787 non-district county-level units with stable 2015–2023 geometry are overlaid on the complete national base map; light gray denotes areas outside the analytical sample, and the inset displays the South China Sea. **(b)** Non-missing coverage for the core county-year variables. The base map uses official administrative-division data approved as GS(2024)0650; thematic overlays are author-generated, and the approved boundary geometry is unchanged. The map does not represent municipal districts or counties undergoing substantial administrative restructuring. This figure uses the official GS(2024)0650 standard-map material solely as the national boundary base layer. The source is the Standard Map Service of the Ministry of Natural Resources of the People's Republic of China (http://bzdt.ch.mnr.gov.cn/), approval number GS(2024)0650.

Hospital and health-center bed availability increased substantially over the study period. Median beds per 10,000 registered residents increased from 35.1 in 2015 to 54.0 in 2023, an increase of 53.8%, and both quartiles shifted upwards (Figure 2a; Table 2, Panel A). This expansion was not accompanied by a comparable compression of relative planning–supply mismatch: its 10th and 90th percentiles changed only from −0.641 and 0.600 in 2015 to −0.615 and 0.600 in 2023 (Figure 2b; Table 2, Panel A). Thus, higher aggregate availability coexisted with persistent within-province differences between territorial planning-context rank and bed-density rank.

**Figure 2:**
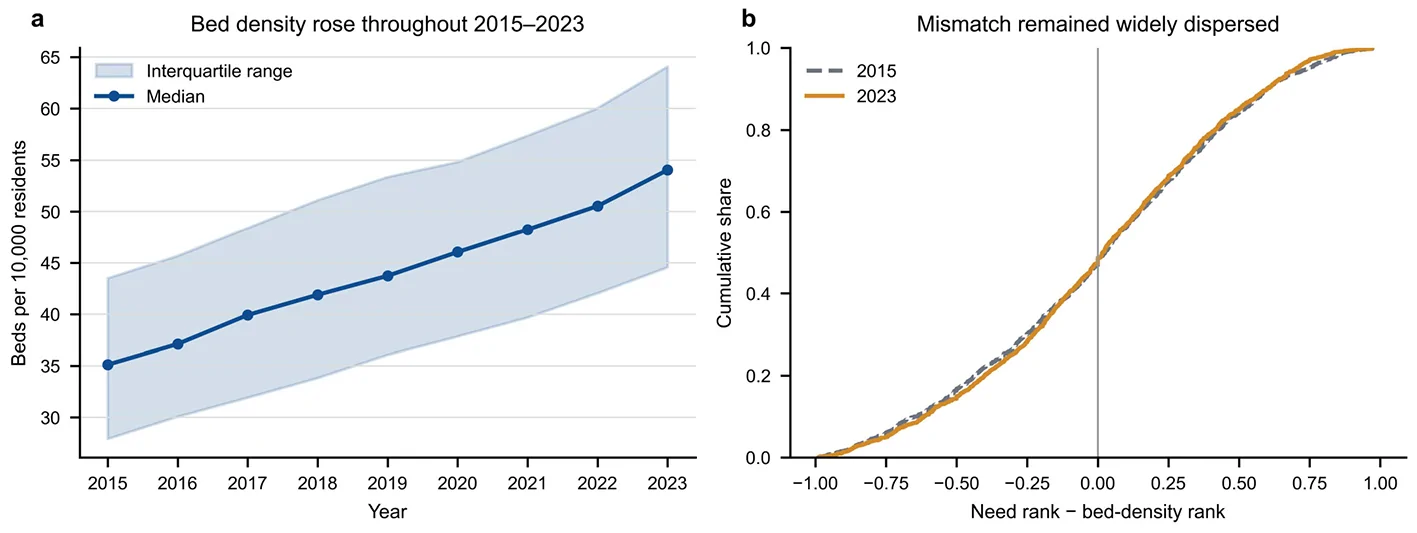
Trends in hospital and health-center bed availability and the distribution of relative planning–supply mismatch. **(a)** Median and interquartile range of beds per 10,000 registered residents from 2015 to 2023. **(b)** Empirical cumulative distributions of the difference between within-province-year territorial planning-context rank and bed-density rank in 2015 and 2023.

Table 2Annual supply, relative mismatch distribution, and high-mismatch-state dynamics.

**Table:** 

Panel A. Annual bed-density and mismatch distribution							
Year	Counties	Median beds/10k	P25 beds/10k	P75 beds/10k	P10 mismatch	P90 mismatch	Shortage
2015	1,787	35.1	27.9	43.5	−0.641	0.600	448
2016	1,787	37.1	30.1	45.7	−0.634	0.601	451
2017	1,787	39.9	31.9	48.4	−0.634	0.594	451
2018	1,787	41.9	33.8	51.1	−0.615	0.598	453
2019	1,787	43.7	36.1	53.4	−0.626	0.595	456
2020	1,787	46.0	37.9	54.8	−0.622	0.585	449
2021	1,787	48.3	39.7	57.4	−0.618	0.583	453
2022	1,787	50.5	42.0	60.0	−0.622	0.598	452
2023	1,787	54.0	44.6	64.1	−0.615	0.600	452

**Table:** 

Panel B. Transition flows and ongoing spell duration					
Year	Entries	Exits	Remain short	Median spell (years)	Current spells ≥3 years
2016	70	67	381	2.0	0
2017	71	71	380	3.0	338
2018	72	70	381	4.0	329
2019	84	81	372	5.0	329
2020	69	76	380	6.0	328
2021	66	62	387	7.0	337
2022	69	70	383	7.0	342
2023	88	88	364	6.0	326

The annual high-mismatch count is mechanically close to one-quarter because the state is defined by the annual upper quartile; small deviations arise from ties and province-specific group sizes. Entries and exits are calculated only when both adjacent-year states are observed. Spell duration counts consecutive years spent in the current high-mismatch state.

### 3.2 Annual bed adjustment: block-level contributions of planning context and capacity

For log total beds, the planning-context-only and capacity-only models produced similar within-*R*^2^ values of 0.0023 and 0.0021, respectively, while the joint model reached only 0.00376. The exact two-block Shapley allocation assigned 53.1% of the explained within-county variation to the planning-context block and 46.9% to the capacity block (Figure 3a; Table 3, Panel A). Because the joint model explained less than 0.4% of within-county variation, these percentages reflect an exploratory allocation of limited model fit rather than substantively large explanatory contributions. Neither block clearly dominated annual adjustment in total-bed stocks. Within the joint component model, fiscal revenue per capita was positively associated with total beds (β = 0.0196, *p* = 0.041; Table 3, Panel B).

**Figure 3:**
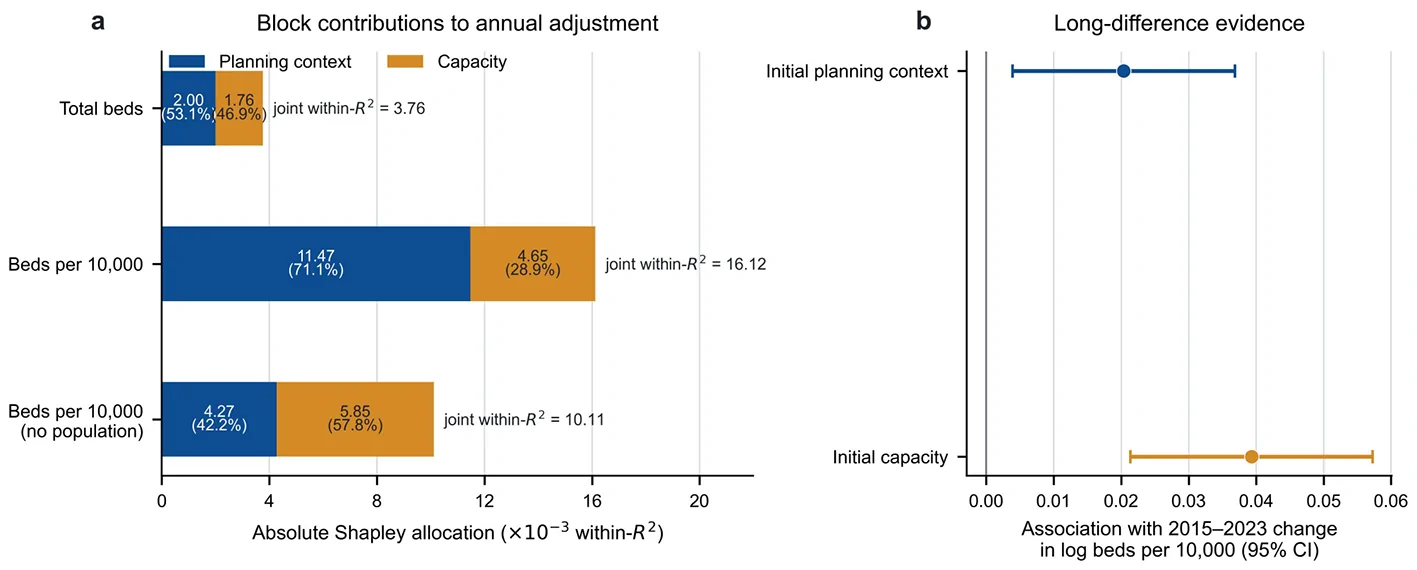
Relative contribution of territorial planning context and local capacity. **(a)** Exact two-block Shapley allocations of joint within-*R*^2^ for total beds and beds per 10,000 residents. Absolute allocations are shown together with percentage shares; the percentages represent exploratory model-fit accounting rather than normative importance. **(b)** Associations of initial planning context and capacity with the 2015–2023 change in log beds per 10,000 residents; points are coefficients, and bars are 95% confidence intervals.

Table 3Block-level contributions of territorial planning context and local capacity to annual bed adjustment.

**Table:** 

Panel A. Block-level model fit and Shapley decomposition						
Outcome	Block	Only-block/joint within-*R*^2^	Unique Δ*R*^2^/absolute Shapley	Shapley share	Joint *F*	Joint block *p*-value
Total beds	Planning context	0.00229	0.00170	53.1%	2.38	0.068
		0.00376	0.001997			
Total beds	Capacity	0.00206	0.00147	46.9%	1.68	0.151
		0.00376	0.001762			
Beds per 10,000	Planning context	0.01463	0.00831	71.1%	11.16	< 0.001
		0.01612	0.011469			
Beds per 10,000	Capacity	0.00781	0.00149	28.9%	1.80	0.127
		0.01612	0.004652			
Beds per 10,000 (no population)	Planning context	0.00623	0.00230	42.2%	2.39	0.092
		0.01011	0.004267			
Beds per 10,000 (no population)	Capacity	0.00781	0.00388	57.8%	4.01	0.003
		0.01011	0.005848

**Table:** 

Panel B. Joint component coefficients		
Component	Log total beds	Log beds per 10,000
Planning context: population	0.111 (0.068)	−0.367^***^ (0.074)
Planning context: dispersion	−0.041 (0.061)	−0.008 (0.068)
Planning context: agriculture	−0.015 (0.012)	−0.017 (0.011)
Capacity: fiscal	0.020^**^ (0.010)	0.020^**^ (0.009)
Capacity: autonomy	−0.007 (0.006)	−0.006 (0.005)
Capacity: GDP	0.020 (0.016)	0.019 (0.015)
Capacity: savings	−0.010 (0.014)	−0.002 (0.012)

All block comparisons use an identical sample, county fixed effects, province-by-year fixed effects, and county-clustered standard errors. Only-block fit, joint within-R^2^, leave-one-block-out ΔR^2^, and absolute Shapley allocations are reported together. Shapley shares quantify exploratory contributions to limited explained within-county variation and do not indicate normative importance or evidence that allocation follows planning context. The no-population planning-context specification excludes population scale from the planning proxy. Standard errors are in parentheses. *p* < 0.10, *p* < 0.05, *p* < 0.01.

For log beds per 10,000 registered residents, the planning-context-only model produced a within-*R*^2^ of 0.0146, compared with 0.0078 for the capacity-only model and 0.01612 for the joint model. The baseline Shapley allocation assigned 71.1% of the explained within-county variation to planning context and 28.9% to capacity. This larger planning-context share did not indicate a normatively aligned allocation because it was driven mainly by the negative population coefficient (β = −0.367, *p* < 0.001). Fiscal revenue per capita was positively associated with bed density (β = 0.0195, *p* = 0.028; Table 3, Panel B).

The population-exclusion specification materially changed the block comparison. When population was removed from the planning-context proxy, the joint bed-density model had a within-*R*^2^ of 0.01011. The capacity block received 57.8% of the exploratory Shapley allocation and was jointly significant (*p* = 0.003), whereas the no-population planning-context block received 42.2% and was only suggestive (*p* = 0.092; Table 3, Panel A). The baseline conclusion that planning context contributed more to the bed-density model was therefore sensitive to the inclusion of population.

### 3.3 Initial conditions and the expansion–alignment paradox, 2015–2023

The long-difference estimates, based on a common sample of 1,764 counties, distinguished aggregate expansion from population-scaled growth. A one-standard-deviation higher 2015 planning-context proxy was associated with greater growth in log total beds (β = 0.0425, *p* < 0.001), whereas initial capacity was negatively associated with total-bed growth (β = −0.0389, *p* < 0.001). For log beds per 10,000 residents, both initial planning context (β = 0.0204, *p* = 0.015) and initial capacity (β = 0.0393, *p* < 0.001) were positively associated with cumulative growth (Figure 3b; Table 4).

**Table 4:** Associations of baseline territorial planning context and capacity with 2015–2023 changes.

Variable	Δ log total beds	Δ log beds per 10,000	Δ mismatch
Initial need	0.043^***^ (0.012)	0.020^**^ (0.008)	0.095^***^ (0.012)
Initial capacity	−0.039^***^ (0.011)	0.039^***^ (0.009)	−0.048^***^ (0.010)
Observations	1,764	1,764	1,764
*R* ^2^	0.217	0.473	0.206

Entries are coefficients with robust standard errors in parentheses. Models include 2015 province fixed effects and the initial level of the dependent variable. The long-difference sample is N = 1,764 for each outcome. *p* < 0.10, *p* < 0.05, *p* < 0.01.

Initial conditions were associated with changes in relative planning–supply mismatch in different ways. Higher initial planning context was associated with a widening of mismatch (β = 0.0955, *p* < 0.001), whereas higher initial capacity was associated with a reduction in mismatch (β = −0.0477, *p* < 0.001; Table 4). Counties with higher planning-context values therefore experienced greater aggregate bed expansion but less favorable movement in relative alignment. By contrast, counties with higher initial capacity experienced slower aggregate expansion but more favorable changes in population-scaled supply and relative mismatch.

This expansion–alignment paradox shows that growth in bed stocks and improvement in relative territorial alignment were distinct empirical margins. The estimates identify conditional associations with initial conditions and do not establish the mechanisms through which capital investment, recurrent financing, workforce recruitment, or local implementation capacity produced these divergent patterns.

### 3.4 Spatial distribution of relative planning–supply mismatch

Relative planning–supply mismatch remained spatially heterogeneous in both endpoint years (Figure 4). Positive and negative mismatch values frequently occurred in adjacent counties, producing a fine-grained territorial mosaic rather than a uniformly disadvantaged macro-region. Because mismatch was constructed from ranks calculated within the same province and year, the mapped pattern describes a county's relative planning–supply position rather than absolute bed sufficiency.

**Figure 4:**
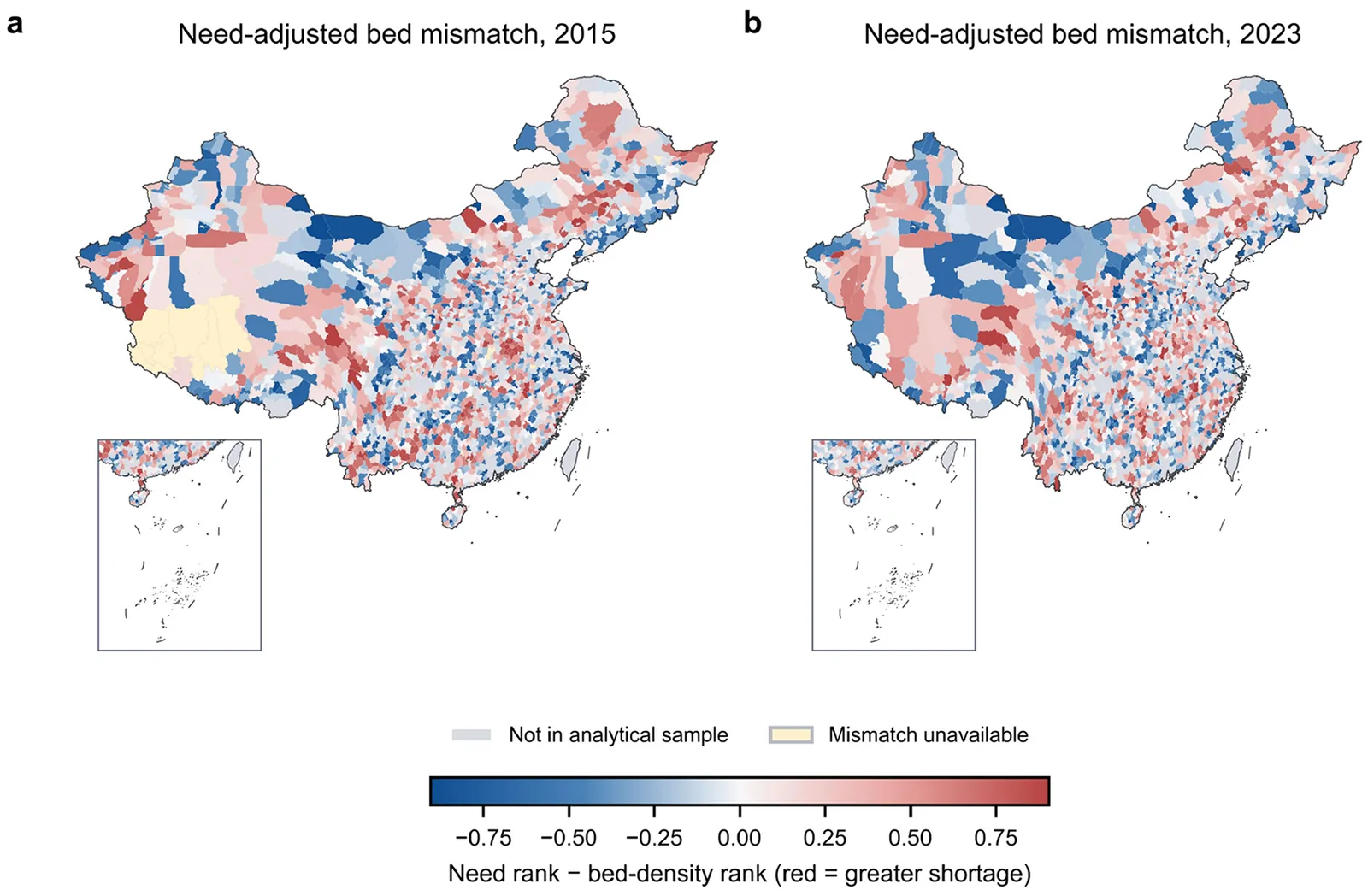
Spatial distribution of relative planning–supply mismatch in 2015 and 2023. Positive values indicate that a county's territorial planning-context rank exceeded its bed-density rank within the same province and year. The measure describes the relative planning–supply position and does not establish an absolute or clinically verified bed shortage. Light gray denotes areas outside the analytical sample, and pale yellow denotes unavailable mismatch values. Insets display the South China Sea. The base map uses official administrative-division data approved as GS(2024)0650; thematic overlays are author-generated and the approved boundary geometry is unchanged. This figure uses the official GS(2024)0650 standard-map material solely as the national boundary base layer. The source is the Standard Map Service of the Ministry of Natural Resources of the People's Republic of China (http://bzdt.ch.mnr.gov.cn/), approval number GS(2024)0650.

The 2015 and 2023 maps also showed that the national increase in bed availability did not eliminate the spatially uneven distribution of relative mismatch. However, the maps alone do not identify whether the observed pattern arose from common policy environments, fiscal conditions, health-professional labor markets, transport constraints, or population mobility. These mechanisms are examined as interpretations in the Discussion section rather than as directly observed results.

### 3.5 Persistent relative mismatch and exit from the high-mismatch state

The corrected 1-year transition matrix contained 14,203 observed transitions. Among 10,600 observations previously outside the high-mismatch state, 10,024 remained outside it (94.6%), and 576 entered the high-mismatch state (5.4%). Among 3,603 observations previously in the high-mismatch state, 575 exited (16.0%), and 3,028 remained in that state (84.0%; Table 5, Panel A). Across the study period, 572 counties were observed in the high-mismatch state in at least 3 years. These results indicate persistence in relative upper-tail membership rather than persistent absolute healthcare-resource shortage.

Table 5High-mismatch-state transitions, persistence, and exit models.

**Table:** 

Panel A. One-year high-mismatch-state transition matrix		
Previous-year state	Current non-shortage	Current shortage
Previous non-shortage	10,024 (94.6%)	576 (5.4%)
Previous shortage	575 (16.0%)	3,028 (84.0%)

**Table:** 

Panel B. Persistence linear-probability models and high-mismatch-state exit logit models				
Variable	(1) Shortage	(2) Shortage	(3) Exit	(4) Exit
Lagged need	0.193^***^ (0.0189)	0.193^***^ (0.0189)	−0.811^***^ (0.111)	−0.861^***^ (0.117)
Lagged capacity	−0.0321^**^ (0.0159)	−0.0342^**^ (0.0174)	0.265^***^ (0.0902)	0.241^**^ (0.121)
Capacity × agriculture-dependent		0.00841 (0.0358)		0.0744 (0.152)
Lagged high-mismatch spell duration			−0.284^***^ (0.0300)	−0.287^***^ (0.0301)
Observations	14,158	14,158	3,591	3,591
Pseudo *R*^2^			0.0943	0.0947
Within *R*^2^	0.0229	0.0229		

The high-mismatch state is defined by the annual upper quartile of relative planning–supply mismatch and does not represent an absolute or clinically verified bed shortage. Panel (A) reports transition counts with row percentages in parentheses, conditional on the previous year's observed state. Models (1)–(2) include county and province-by-year fixed effects; models (3)–(4) include year and province indicators and are estimated among county-year observations in the high-mismatch state in the preceding year. County-clustered standard errors are in parentheses. *p* < 0.05 and *p* < 0.01.

In the baseline exit logit model, higher lagged planning context was associated with a lower probability of exit (β = −0.811, *p* < 0.001), whereas higher lagged capacity was associated with a higher probability of exit (β = 0.265, *p* = 0.003). Longer prior high-mismatch spell duration was also negatively associated with exit (β = −0.284, *p* < 0.001; *N* = 3, 591; Table 5, Panel B). The interaction between capacity and high baseline agricultural share was not statistically significant.

Average adjusted probabilities translated these associations into substantive magnitudes (Figure 5a; Table 6, Panel C). At mean planning context and a 1-year high-mismatch spell, the adjusted probability of exit increased from 34.3% at capacity one standard deviation below the mean to 43.5% at capacity one standard deviation above the mean. At a 5-year spell duration, the corresponding probabilities were 16.0% and 22.1%. Figure 5b shows the distribution of consecutive high-mismatch spell lengths.

**Figure 5:**
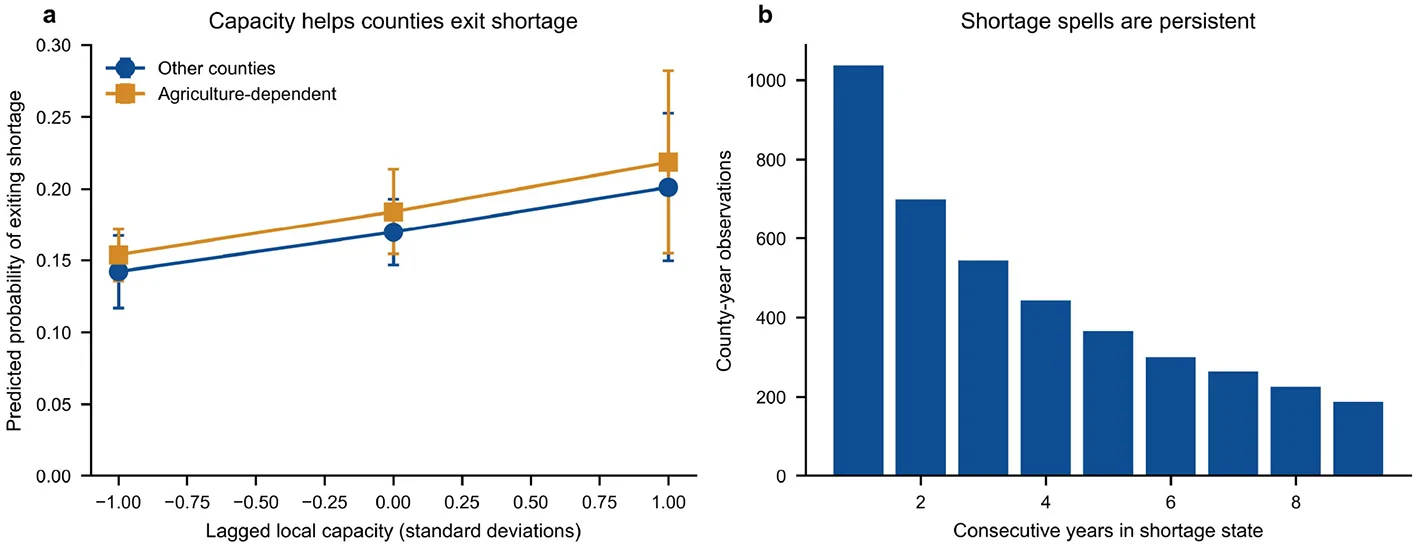
Persistent relative mismatch and adjusted exit probabilities. **(a)** Average adjusted probabilities of exiting the high-mismatch state at selected values of lagged local capacity; bars are 95% confidence intervals. **(b)** Distribution of consecutive years spent in the high-mismatch state. The probabilities are adjusted associations rather than causal counterfactuals.

Table 6Component diagnostics and adjusted probabilities of exiting the high-mismatch state.

**Table:** 

Panel A. Pooled component correlations							
Component	Need: population	Need: dispersion	Need: agriculture	Capacity: fiscal	Capacity: autonomy	Capacity: GDP	Capacity: savings
Need: population	1.000						
Need: dispersion	−0.834	1.000					
Need: agriculture	−0.094	0.244	1.000				
Capacity: fiscal	−0.123	0.047	−0.519	1.000			
Capacity: autonomy	0.318	−0.375	−0.523	0.786	1.000		
Capacity: GDP	−0.100	0.028	−0.514	0.809	0.656	1.000	
Capacity: savings	0.128	−0.255	−0.417	0.613	0.573	0.645	1.000

**Table:** 

Panel B. Common-sample component diagnostics					
Component	County-years	VIF	Within-county SD	Between-county SD	Within variance share
Need: population	15,910	3.77	0.045	0.998	0.2%
Need: dispersion	15,910	4.17	0.036	1.002	0.1%
Need: agriculture	15,910	1.62	0.261	0.958	6.9%
Capacity: fiscal	15,910	6.50	0.301	0.954	9.0%
Capacity: autonomy	15,910	5.21	0.357	0.933	12.8%
Capacity: GDP	15,910	3.42	0.254	0.965	6.5%
Capacity: savings	15,910	2.10	0.192	0.984	3.7%

**Table:** 

Panel C. Average adjusted probabilities of exiting the high-mismatch state by local capacity and high-mismatch spell duration					
Lagged capacity	1-year spell	3-year spell	5-year spell
−1 SD	34.3% [30.1, 38.5]	24.0% [20.9, 27.2]	16.0% [13.0, 19.0]
0 SD	38.8% [35.0, 42.6]	27.8% [24.6, 31.0]	18.9% [15.5, 22.3]
+1 SD	43.5% [37.4, 49.6]	31.9% [26.3, 37.5]	22.1% [16.8, 27.4]

Panel B: Pairwise Pearson correlations use the common complete component sample. The lower triangle is shown. VIF denotes the variance inflation factor. The within variance share is within-SD squared divided by the sum of within-SD squared and between-SD squared; it is a descriptive diagnostic rather than a model R-squared. Cells report adjusted probabilities with 95% confidence intervals in brackets. Probabilities are calculated holding lagged need at its standardized mean and averaging over the observed year and province indicators in the baseline exit logit estimation sample. They are associational, not causal counterfactuals.

These adjusted probabilities describe conditional associations rather than causal counterfactuals. In particular, counties observed in longer spells are selected survivors that did not exit in preceding periods. The lower exit probability associated with longer spell duration therefore cannot, by itself, establish causal cumulative disadvantage.

### 3.6 Spatial dependence and prefecture-border conditions

Global Moran's *I* was positive and permutation-significant in every year, decreasing descriptively from 0.276 in 2015 to 0.221 in 2023 (all permutation *p* = 0.001; Figure 6a; Table 7, Panel A). Local analysis identified 178 high–high and 131 low–low relative mismatch counties in 2015, compared with 173 high–high and 95 low–low counties in 2023 (Table 7, Panel C). Relative planning–supply mismatch therefore remained geographically structured throughout the study period.

**Figure 6:**
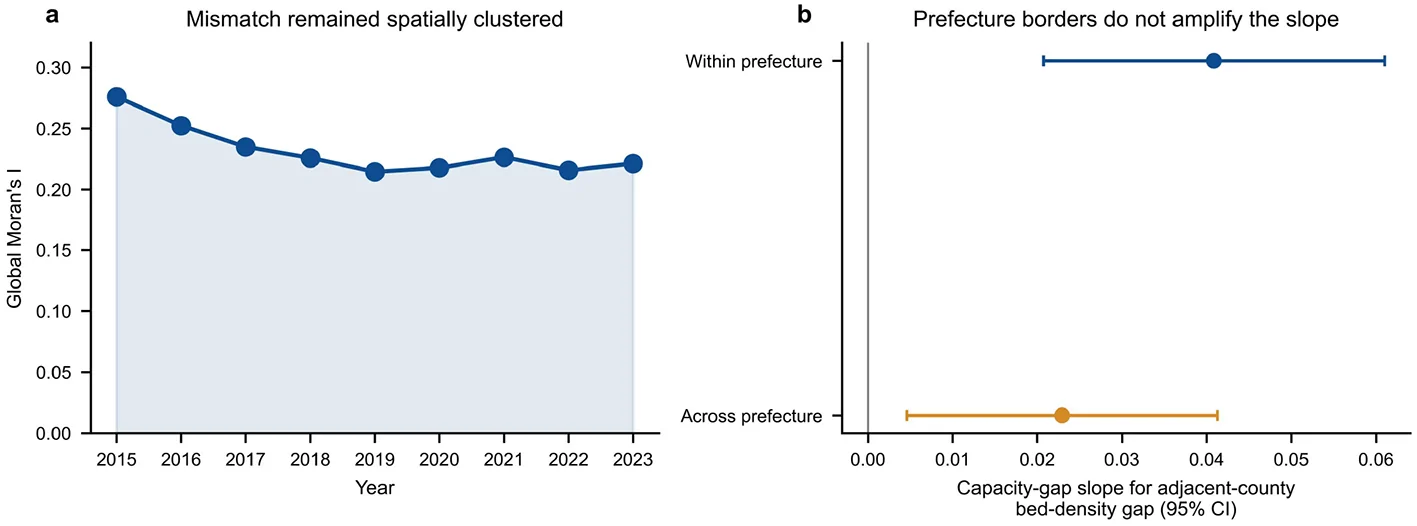
Spatial clustering and adjacent-county capacity gradients. **(a)** Annual Global Moran's *I* for relative planning–supply mismatch; all permutation *p*-values were 0.001. **(b)** Capacity-gap associations for adjacent counties within and across prefecture boundaries; bars are 95% confidence intervals. The directional analysis showed that the higher-capacity county had higher bed density in 63.3% of adjacent pair-years, while the directional cross-prefecture interaction was not statistically significant.

Table 7Spatial autocorrelation, LISA classifications, and spatial fixed-effects estimates.

**Table:** 

Panel A. Annual Global Moran's *I*					
Year	*N*	Moran's *I*	Expected *I*	Permutation *p*	*z*
2015	1,761	0.276; 0.251	−0.0006	0.001	16.60
2016	1,763	0.252; 0.236	−0.0006	0.001	14.80
2017	1,771	0.235; 0.228	−0.0006	0.001	14.89
2018	1,774	0.226; 0.223	−0.0006	0.001	13.93
2019	1,775	0.215; 0.220	−0.0006	0.001	13.36
2020	1,757	0.218; 0.231	−0.0006	0.001	12.94
2021	1,775	0.226; 0.231	−0.0006	0.001	13.92
2022	1,770	0.216; 0.220	−0.0006	0.001	13.45
2023	1,775	0.221; 0.224	−0.0006	0.001	13.48

**Table:** 

Panel B. Spatial fixed-effects lag model						
Term	Coefficient	SE	*z*	*p*-value	Counties	County-years
Planning context (province-by-year FE)	−0.0889^***^	0.0156	−5.70	< 0.001	1,620	14,580
Capacity index (province-by-year FE)	0.0615^***^	0.0074	8.28	< 0.001	1,620	14,580
Spatial lag (rho), province-by-year FE	0.0903^***^	0.0112	8.06	< 0.001	1,620	14,580

**Table:** 

Panel C. Endpoint LISA classification		
LISA cluster	2015	2023
Not significant	1,360	1,428
High–high relative mismatch	178	173
Low–low relative mismatch	131	95
Low–high spatial outlier	61	45
High–low spatial outlier	31	34

Panel (A) uses 999 random permutations and row-standardized Queen-contiguity weights. Moran's I is shown for the baseline and no-population planning-context mismatch definitions; all permutation p-values are 0.001. The preferred spatial panel includes county and province-by-year fixed effects, with a national-year fixed-effects specification shown for comparison. The spatial-lag coefficient is interpreted as spatial association, not a causal spillover. High–high and low–low denote relative mismatch clusters; high–low and low–high denote spatial outliers. These classifications do not identify patient flows or causal spillovers. *p* < 0.10, *p* < 0.05, and *p* < 0.01.

The spatial-panel results changed substantially after temporal effects were introduced. In the specification with county and national-year fixed effects, the spatial-lag coefficient was ρ = 0.214 (*p* < 0.001). In the preferred specification with county and province-by-year fixed effects, planning context was negatively associated with log beds per 10,000 residents (β = −0.0889, *p* < 0.001), capacity was positively associated with bed density (β = 0.0615, *p* < 0.001), and the spatial-lag coefficient declined to ρ = 0.0903 (*p* < 0.001; Table 7, Panel B). The former estimate of ρ = 0.610, obtained without adequate temporal effects, was not retained because it partly absorbed common temporal trends. The revised spatial coefficients are interpreted as conditional spatial associations rather than causal spillovers.

Adjacent-county pair models using multiway clustering on both county endpoints showed that larger absolute capacity gaps were associated with larger bed-density gaps (β = 0.0409, *p* < 0.001; Table 8, Panel A). The cross-prefecture interaction was negative but not statistically significant (β = −0.0179, *p* = 0.229), providing no evidence of an additional average prefecture-border penalty.

Table 8Adjacent-county capacity differences, directional associations, and prefecture-boundary conditions.

**Table:** 

Panel A. Pair fixed-effects estimates			
Variable	(1) Bed-density gap	(2) Mismatch gap	(3) Bed-density gap
Planning-context gap	0.0292 (0.0180)	0.413^***^ (0.0196)	
Capacity gap	0.0409^***^ (0.0114)	0.0260^**^ (0.0112)	0.0714^***^ (0.0175)
Cross-prefecture interaction	−0.0179 (0.0149)	0.0198 (0.0151)	0.0095 (0.0240)
Higher-capacity endpoint has higher bed density			63.3% of pair-years
Observations	36,551	36,551	36,551
Within *R*^2^	0.0020	0.0437	0.0042

**Table:** 

Panel B. Capacity-gap slopes by border condition				
Border condition	Slope	SE	*p*-value	95% CI
All adjacent pairs	0.0714	0.0175	< 0.001	[0.037, 0.106]
Cross-prefecture interaction	0.0095	0.0240	0.694	[−0.037, 0.056]

Pair and year fixed effects are included. Standard errors are multiway clustered on both county endpoints because counties may appear in multiple adjacent pairs. Absolute differences are used in the first two specifications; the directional specification compares the higher-capacity endpoint with the lower-capacity endpoint. These estimates are border-conditioned associations and are not a geographic regression-discontinuity design. *p* < 0.10, *p* < 0.05, and *p* < 0.01.

The directional specification provided a more direct comparison of the two endpoints. The higher-capacity county also had higher bed density in 63.3% of adjacent pair-years. A one-unit capacity advantage was associated with a 0.0714-log-point bed-density advantage (*p* < 0.001; Figure 6b; Table 8, Panel B). The directional cross-prefecture interaction was small and not statistically significant (β = 0.0095, *p* = 0.694). The null interactions do not demonstrate that administrative borders are irrelevant; they indicate that capacity gradients were more consistent than an additional average prefecture-border effect in these specifications.

### 3.7 Robustness, diagnostics, and substantive magnitudes

#### 3.7.1 Robustness to alternative definitions and samples

The positive capacity coefficient in the baseline high-mismatch-exit model was generally reproduced under alternative percentile thresholds and mismatch constructions. The coefficient was 0.265 under the baseline top-25% definition (*p* = 0.003), 0.204 under the top-20% definition (*p* = 0.039), and 0.255 under the top-30% definition (*p* = 0.001). It was 0.163 under the population-weighted planning-context definition (*p* = 0.058), 0.173 under the dispersion-weighted definition (*p* = 0.023), 0.238 under the no-population definition (*p* = 0.001), and 0.175 under the regression-residual definition (*p* = 0.005; Table 9, Panel A).

**Table 9:** Robustness to alternative mismatch definitions, outcomes, and sample restrictions.

Panel A. Alternative high-mismatch-state definitions				
Definition	Capacity coefficient	SE	*p*-value	*N*
Baseline: rank mismatch, 25%	0.265^***^	0.0902	0.003	3,591
Threshold: top 20%	0.204^**^	0.0987	0.039	2,878
Threshold: top 30%	0.255^***^	0.0781	0.001	4,306
Planning-context weights: population 50%	0.163^*^	0.0860	0.058	3,568
Planning-context weights: dispersion 50%	0.173^**^	0.0759	0.023	3,601
No-population planning-context mismatch	0.238^***^	0.0751	0.001	3,539
Planning-context-model residual mismatch	0.175^***^	0.0629	0.005	3,544
HRDI supply-rank mismatch	0.233^**^	0.1002	0.020	3,600
Need-model residual mismatch	−0.233^**^	0.1002	0.020	3,600
**Panel B. Annual bed-adjustment outcome and sample sensitivity**				
**Specification**	**Lagged need**	**Lagged capacity**	***N***	**Within *R*^2^**
Total beds, baseline	−0.027^**^ (0.014)	0.009 (0.010)	14,176	0.0014
Bed density, winsorized 1%	−0.032^**^ (0.013)	0.039^***^ (0.011)	14,158	0.0051
Bed density, untrimmed	−0.032^**^ (0.014)	0.040^***^ (0.012)	14,158	0.0046
Exclude population < 20,000	−0.035^**^ (0.014)	0.037^***^ (0.012)	14,044	0.0045
Exclude bed-density tails	−0.022^*^ (0.012)	0.040^***^ (0.010)	13,913	0.0046
HRDI-adjusted beds	−0.031^**^ (0.013)	0.029^***^ (0.010)	14,158	0.0035
**Panel C. Agreement across mismatch definitions**				
**Definition**	**County-years**	**Correlation**	**High-mismatch-state agreement**	**High-mismatch count**
Equal weights (baseline)	16,022	1.000	100.0%	4,065
Population weight 50%	16,022	0.944	92.3%	4,043
Dispersion weight 50%	16,022	0.943	91.3%	4,076
No-population planning-context mismatch	16,022	0.870	86.9%	4,011
HRDI supply rank	16,022	0.781	82.0%	4,080
Need-model residual	16,022	0.558	73.8%	4,035

Panel (A) reports lagged capacity coefficients from high-mismatch-state exit logit models. Every model controls for the corresponding lagged planning-context measure, lagged high-mismatch spell duration, year indicators, and province indicators. Entries are coefficients with county-clustered standard errors in parentheses. Panel (C) reports correlations and high-mismatch-state agreement against the equal-weight rank-mismatch baseline. The high-mismatch state is a relative upper-tail classification rather than an absolute clinical shortage standard; the HRDI reversal is retained as evidence of construct sensitivity. *p* < 0.10, *p* < 0.05, and *p* < 0.01

The HRDI supply-rank specification produced a negative capacity coefficient (β = −0.233, *p* = 0.020). The capacity association was therefore generally positive across percentile thresholds, alternative weights, the no-population proxy, and the residual construction, but it was not invariant to the definition of geographically adjusted supply adequacy.

Removing population from the planning-context proxy produced a mismatch measure that correlated with the baseline measure at 0.870, and agreement in high-mismatch-state classification was 86.9%. Persistence among observations previously in the no-population high-mismatch state was 81.4%, compared with 84.0% under the baseline definition, while the corresponding exit rate was 18.6%. Thus, persistence remained substantial after population exclusion but was not completely invariant to the indicator construction.

Spatial sensitivity results were similar. Global Moran's *I* for the no-population mismatch remained significant in all 9 years, with *I* = 0.251 in 2015 and *I* = 0.224 in 2023 and permutation *p* = 0.001 throughout. Baseline and no-population Local Indicators of Spatial Association (LISA) classifications agreed for 84.8% of counties in both 2015 and 2023.

The annual capacity association with bed density remained positive after use of untrimmed outcomes, exclusion of counties with fewer than 20,000 registered residents, removal of the upper and lower 1% of bed-density observations and replacement of the outcome with HRDI-adjusted bed supply (Table 9, Panel B).

#### 3.7.2 Annual dynamics and component diagnostics

Component diagnostics showed substantial overlap within both the planning-context and capacity blocks. The correlation between population scale and geographic dispersion was −0.834, while several fiscal-economic capacity components were correlated at approximately 0.6–0.8. The maximum variance inflation factor was 6.50 for the fiscal component. The within-county variance shares were only 0.2% for population scale and 0.1% for geographic dispersion, confirming that their fixed-effects coefficients were identified from limited year-to-year variation (Table 6, Panels A–B).

The pooled PCA provided a construct-sensitivity assessment. The first planning-context principal component explained 63.5% of component variance but correlated only 0.298 with the equal-weight planning-context proxy because it primarily contrasted population scale with dispersion. By contrast, the first capacity principal component explained 76.2% of the component variance and had a correlation of 0.9998 with the equal-weight capacity index. In the PCA-based bed-density model, the capacity coefficient remained positive (β = 0.0449, *p* < 0.001; Table 6).

The Mundlak decomposition distinguished limited within-county changes from persistent between-county differences. For bed density, the within-county planning-context coefficient was −0.0792 (*p* = 0.020), and the within-county capacity coefficient was 0.0432 (*p* = 0.003). The between-county planning-context coefficient was 0.0459 (*p* = 0.056), whereas the between-county capacity coefficient was 0.1866 (*p* < 0.001; Table 6). Persistent cross-county capacity differences were therefore substantially stronger than the corresponding within-county changes, reinforcing the need for caution when interpreting the fixed-effects component estimates.

#### 3.7.3 Adjusted exit probabilities

Pandemic-period interactions showed no detectable change in the planning-context association. Relative to 2016–2019, the planning-context slope difference was not significant in 2020–2022 (*p* = 0.957) or in 2023 (*p* = 0.709). By contrast, the capacity association with bed-density adjustment was weaker during 2020–2022 by 0.0191 (*p* = 0.003) and in 2023 by 0.0374 (*p* < 0.001; Table 6). These estimates indicate period-specific attenuation of the observed capacity association but do not identify a causal pandemic effect.

The discrete-time complementary log–log model supported the baseline exit association. Higher lagged capacity was associated with a greater conditional exit hazard, with a hazard ratio of 1.232 (*p* = 0.006). Relative to a 1-year high-mismatch spell, the exit hazard ratios were 0.587 for 2-year spells, 0.397 for 3-year spells, 0.308 for 4-year spells and 0.258 for spells of 5 or more years (Table 6). The 5-or-more-year estimate was statistically significant (*p* < 0.001).

The duration pattern indicates that counties observed in longer high-mismatch spells had lower conditional exit hazards. However, these counties are selected survivors that did not exit in previous periods. The duration coefficients therefore do not establish causal duration dependence or cumulative disadvantage.

## 4 Discussion

### 4.1 Principal findings

This study does not identify a simple winner between territorial planning context and local fiscal-economic capacity. Instead, the two constructs were associated with different margins of county health-system adjustment. For annual total-bed stocks, the planning-context and capacity blocks received broadly similar shares of the limited within-county variation explained by the joint model. For beds per 10,000 registered residents, the baseline Shapley decomposition assigned a larger share to the planning-context block, but this allocation was driven mainly by the negative population coefficient and occurred within a model with modest absolute explanatory power. When population was removed from the planning-context proxy, the capacity block received the larger share and remained jointly significant. The Shapley results therefore provide exploratory model-fit accounting rather than definitive evidence that either block is substantively dominant.

The longer-term estimates revealed a more theoretically informative divergence. Counties with higher initial planning-context values experienced greater growth in total beds and beds per 10,000 residents, but also less favorable changes in relative planning–supply mismatch. Counties with higher initial capacity experienced slower aggregate expansion but more favorable population-scaled growth, lower relative mismatch, and better prospects of exiting the baseline high-mismatch state. Aggregate expansion, improvement in relative alignment, and recovery from persistent relative mismatch were therefore distinct system outcomes.

The dynamic evidence reinforced this distinction. Among county-years previously in the high-mismatch state, 84.0% remained in that state in the following year, whereas only 16.0% exited. A total of 572 counties occupied the high-mismatch state in at least three study years. These figures describe persistence in relative upper-tail membership rather than persistent absolute healthcare-resource shortage. Spatial clustering also remained statistically detectable, but the preferred spatial coefficient declined markedly after province-by-year effects were introduced. Adjacent-county models showed a consistent capacity gradient, including higher bed density in the higher-capacity county in 63.3% of pair-years, but did not identify an additional average prefecture-border penalty.

### 4.2 The expansion–alignment paradox and the role of local capacity

Total beds, beds per 10,000 residents and the relative planning–supply mismatch address related but non-equivalent planning questions. Total beds measure the aggregate scale of a county's bed stock, whereas bed density also varies with the registered population. Relative mismatch compares a county's planning-context rank with its bed-density rank within the same province and year. A county may therefore add beds while losing beds per resident if population grows more rapidly, and it may remain in approximately the same relative position if comparable counties also expand. Conversely, a county with limited additions may experience an increase in bed density when its registered population declines. Previous studies similarly demonstrate that evaluations of health-resource equity and efficiency depend on whether resources are assessed at the population, geographic area, income, utilization, or institutional level (16, 30–34).

This distinction is essential for interpreting the baseline block decomposition. The larger planning-context share in the original bed-density model does not establish that allocation decisions responded normatively to healthcare need. Population enters both the planning-context proxy and the denominator of bed density, creating a partly mechanical relationship. The negative population coefficient may reflect bed additions that did not keep pace with population growth, density gains in counties with declining registered populations, or both. After population was excluded, capacity received 57.8% of the exploratory Shapley allocation. The no-population planning-context block was only suggestive, whereas the capacity block remained jointly significant. Accordingly, the appropriate conclusion is not that the system followed need, but that demographic scale was central to population-scaled adjustment and that the relative block allocation was specification sensitive.

The long-difference results reveal an expansion–alignment paradox. Higher initial planning-context values were associated with greater aggregate bed growth but a wider relative mismatch, whereas higher initial capacity was associated with slower total-bed growth but more favorable bed-density growth and relative alignment. One possible interpretation is that population scale and territorial service obligations are more visible in capital-planning formulas, allowing counties with stronger planning pressures to receive additional nominal capacity. However, translating bed additions into sustained population-scaled service capacity may require recurrent financing, workforce recruitment, equipment procurement, facility management, and organizational continuity. Counties with stronger fiscal-economic capacity may be better placed to complete this operational conversion even when their absolute additions are smaller.

This explanation is consistent with previous evidence linking county bed availability to government revenue and economic conditions and showing that national or regional resource growth does not necessarily eliminate territorial inequality (15–19, 30–32). It also provides a plausible systems interpretation of why higher-planning-context counties expanded more but did not necessarily improve their relative position. Nevertheless, the present models do not observe investment decisions, staffing processes, facility utilization, or transfer implementation. Capital expansion and operational conversion should therefore be treated as testable mechanisms consistent with the observed pattern, not as causal processes established by the coefficients.

The PCA and Mundlak analyses further qualify the block-level interpretation. The capacity principal component closely reproduced the equal-weight capacity index, whereas the planning principal component correlated only modestly with the equal-weight proxy because it largely contrasted population scale with geographic dispersion. The Mundlak estimates showed that persistent between-county capacity differences were substantially stronger than short-run within-county changes. Population and dispersion also exhibited very limited within-county variation. Fixed-effects estimates for these components are therefore identified from small year-to-year deviations and may be sensitive to measurement error or changes in reporting. These diagnostics favor a cautious interpretation of individual component coefficients and greater emphasis on the convergence of evidence across annual, long-difference, and construct-sensitivity models.

### 4.3 Persistent relative mismatch and the conditions for recovery

The transition analysis converts a cross-sectional allocation measure into a dynamic system state. Because the annual high-mismatch threshold identifies the upper quartile of the relative mismatch distribution, the number of high-mismatch counties is approximately one-quarter of the valid observations. The substantive evidence is not the annual count itself, but the asymmetry between entry and persistence. Only 5.4% of observations previously outside the high-mismatch state entered it, whereas 84.0% of those previously inside remained there. Annual maps can therefore obscure the distinction between temporary rank fluctuations and repeated occupation of an unfavorable relative position.

Local capacity was positively associated with exit from the baseline high-mismatch state, and the no-population specification yielded a similar result. In the discrete-time model, a one-unit increase in lagged capacity was associated with an exit hazard ratio of 1.232. This finding is consistent with the possibility that recurrent finance, workforce recruitment, procurement, and administrative implementation support recovery after a county falls into the high-mismatch tail. It does not imply that fiscal resources alone are sufficient, nor does it establish that increasing fiscal capacity would causally produce exit.

Longer observed spells were associated with progressively lower conditional exit hazards. Counties in spells of 5 or more years had an estimated exit hazard ratio of 0.258 relative to 1-year spells. This pattern should not be interpreted directly as proof that each additional year causes further disadvantage. Counties observed in longer spells are selected survivors that did not exit in earlier periods, and they may differ in unobserved geography, inherited infrastructure, political priorities, provider markets, or administrative capability. The duration results therefore identify conditional persistence rather than causal dependence on duration. A stronger design would require discrete policy timing, richer institutional covariates, or models that separately address unobserved heterogeneity.

The HRDI specification provides an important boundary for the recovery argument. Capacity was positively associated with exit under the baseline, alternative-threshold, alternative-weight, no-population, and residual constructions but negatively associated with exit when supply was ranked using HRDI. HRDI assigns a stronger normative role to geographic area, thereby altering the interpretation of adequate supply in large, sparsely populated counties (16). This reversal should not be treated as a failed robustness check or suppressed as an anomalous result. Instead, it shows that the conclusion about recovery depends partly on whether geographic dispersion is represented on the planning-context side, the supply side, or both.

The policy implication, therefore, is not to replace territorial planning criteria with fiscal-capacity criteria. Counties in persistent high-mismatch states may require multi-year packages combining capital transfers, recurrent financing, workforce support, and implementation assistance. China's experience with public-health equalization similarly indicates that intergovernmental finance can expand coverage, while implementation remains dependent on multilevel funding and local delivery arrangements (33). Monitoring systems should track entry, exit, and spell duration and diagnose whether persistent relative mismatch reflects insufficient capital, inadequate staffing, weak recurrent finance, population change, or a planning measure poorly matched to local geography.

### 4.4 Spatial structure, functional connections, and administrative boundaries

Relative planning–supply mismatch remained spatially organized throughout 2015–2023. Positive Global Moran's *I* and persistent high–high and low–low clusters are consistent with previous evidence that county health resources are geographically concentrated (15–17, 28, 29). The no-population mismatch produced significant Moran's *I* in every year, and its endpoint LISA classifications agreed with the baseline classifications for 84.8% of counties. The spatial pattern is therefore not solely a mechanical product of placing population in both the planning-context proxy and the denominator of bed density.

The magnitude of conditional spatial dependence was nevertheless sensitive to temporal controls. The spatial-lag coefficient was 0.214 when national-year effects were included and 0.090 when province-by-year effects were included. The preferred estimate therefore indicates only a modest residual spatial association after accounting for province-specific annual shocks. The reduction in the coefficient suggests that part of the original spatial association reflected common temporal and regional processes. Spatial dependence should consequently not be described as strong causal spillover or evidence that neighboring counties compensate for one another's bed deficits.

Several mechanisms could generate the remaining spatial structure. Neighboring counties may be exposed to common provincial transfer rules, regional development strategies, hospital-planning practices, or macroeconomic shocks. They may compete for the same health-professional labor market or face shared transport corridors and topographic constraints. Administrative learning and imitation could also diffuse facility-planning decisions across neighboring jurisdictions. These mechanisms produce different empirical predictions and cannot be distinguished using contiguity weights and bed stocks alone. Patient-origin data, referral records, travel-time networks, workforce movements, and policy-timing information would be required to assess them.

The adjacent-county results provide more direct evidence of a capacity gradient. Larger capacity gaps were associated with larger bed-density gaps, and the directional model indicated that higher-capacity counties tended to have higher bed density. However, neither the absolute-gap interaction nor the directional interaction indicated a statistically significant additional prefecture-border penalty. This does not demonstrate that administrative boundaries are irrelevant. Boundaries may influence reimbursement, budgeting, referrals, or patient movement through mechanisms that are not captured by annual differences between adjacent counties. The result indicates only that capacity differences were more consistently associated with bed-density differences than prefecture separation itself.

Recent Chinese evidence also indicates that functional economic and mobility relationships can extend across administrative boundaries and that administrative fragmentation does not necessarily coincide with functional spatial separation (35, 36). These studies do not directly measure healthcare utilization or patient flows, but they support the distinction between administrative adjacency and functional connectivity. Coordinated planning should therefore be based on observed spatial clusters, travel conditions, referral relationships and patient flows rather than on the assumption that every prefecture boundary generates an equivalent degree of friction. Accessibility research likewise shows that facility supply, competing demand, travel impedance, topography, and service hierarchy jointly shape effective access (2–4, 11–14).

### 4.5 Policy implications, limitations, and future research

Three policy implications follow. First, aggregate bed targets should be evaluated alongside population-scaled supply and relative territorial alignment. Planning formulas should incorporate population scale, settlement dispersion, existing supply, direct indicators of health-service need where available, and prior high-mismatch spell duration. The planning-context proxy used here can support territorial monitoring, but it should not substitute for morbidity, aging, or utilization data. Second, capital investment should be linked to recurrent and operational support. Lower-capacity counties may require predictable transfers, workforce programs, procurement assistance, and management support in addition to construction finance. Third, monitoring systems should report entry into the high-mismatch state, exit from it, and spell duration, rather than relying exclusively on annual bed counts.

Spatial coordination is also warranted, but contiguity alone is insufficient to define functional service regions. High–high relative mismatch clusters can identify areas for coordinated assessment, while travel time, referral relationships, and patient-origin data are needed to determine whether neighboring facilities are complementary, substitutable, or jointly inadequate. Broader evidence shows that medical-system and payment reforms can alter resource-allocation efficiency, bed utilization, and the regional distribution of inpatient activity (34, 37, 38). Efficiency-oriented reforms should therefore be accompanied by explicit monitoring of territorial access and relative alignment.

Several limitations qualify the findings. First, the territorial planning-context proxy combines population scale, geographic dispersion, and agricultural structure but does not include direct county-level measures of aging, morbidity, mortality, chronic disease prevalence, or realized healthcare utilization. Agricultural share represents the rural production and service-delivery context rather than a direct healthcare need. Second, population appears in both the baseline planning-context proxy and the denominator of bed density. The no-population results show that the central persistence and spatial findings are not solely mechanical, but the incomplete agreement appropriately limits claims of construct invariance.

Third, the estimates remain vulnerable to reverse causality and omitted confounding. Bed expansion may influence local economic activity and fiscal conditions, while political cycles, bureaucratic incentives, land availability, transfer formulas, policy designation, and inherited infrastructure may affect both capacity and bed allocation. County and province-by-year fixed effects reduce some sources of confounding but do not generate exogenous variation. Fourth, the 2015–2023 period includes the COVID-19 pandemic. The capacity association weakened during 2020–2022 and in 2023, whereas the planning-context association did not change significantly. These interactions indicate period-specific modifications in the observed associations but do not identify a causal pandemic effect.

Future research should integrate bed stocks with workforce, utilization, occupancy, service quality, amenable mortality, age structure, morbidity, travel time, and patient-origin data. Age- and disease-specific benchmarks would provide more direct measures of healthcare need, while referral networks would enable analysis of functional service regions. Clearly timed changes to transfer formulas, hospital planning, administrative boundaries, or payment systems could support stronger causal designs. Within the limits of the present associational design, territorial health-system sustainability requires separate evaluation of capacity expansion, relative planning–supply alignment, and recovery from persistent high-mismatch states.

## 5 Conclusion

Across 1,787 geometry-stable non-district Chinese counties between 2015 and 2023, the median number of hospital and health-center beds per 10,000 registered residents increased substantially, while the distribution of relative planning–supply mismatch changed little. Among county-years in the high-mismatch state in the previous year, 84.0% remained in that state the following year, indicating persistent relative upper-tail membership rather than persistent absolute shortage. Territorial planning context and local fiscal-economic capacity were associated with different dimensions of adjustment. The baseline planning-context block received a larger share of the limited within-county variation in bed density, but this result was driven largely by population scale and was reversed in the relative block ranking when population was excluded. Over the longer term, higher initial planning context was associated with greater aggregate bed expansion but widening relative mismatch, whereas higher initial capacity was associated with more favorable population-scaled growth, lower mismatch, and better prospects of exiting the baseline high-mismatch state. Relative mismatch remained spatially clustered after population exclusion, but the spatial coefficient declined substantially after province-by-year temporal controls were introduced, and prefecture boundaries did not significantly amplify capacity-related bed-density gaps.

These findings support a multi-stage territorial planning strategy that separates aggregate expansion, relative alignment, and recovery from persistent high-mismatch states. Allocation formulas should combine population scale, settlement dispersion, existing supply, direct health-need indicators where available, and prior duration of high mismatch. Counties with limited fiscal-economic capacity may require predictable capital transfers, along with recurrent financing, workforce, and operational support. Monitoring systems should track entry, exit, and spell duration rather than relying exclusively on annual bed counts. Functional service regions should be assessed using travel time, referral relationships, and patient flows rather than contiguity alone. The conclusions remain associational and apply specifically to hospital and health-center beds in geometry-stable non-district counties. The planning-context proxy is not a direct measure of morbidity-based need, relative mismatch does not define clinically sufficient supply, and bed stocks do not establish equity in utilization or health outcomes. Future research should therefore integrate bed capacity with workforce, utilization, quality, morbidity, mortality, and functional accessibility data.

## Data Availability

The original contributions presented in the study are included in the article/supplementary material, further inquiries can be directed to the corresponding author.

## References

[1] Braveman PA Kumanyika S Fielding J Laveist T Borrell LN Manderscheid R . Health disparities and health equity: the issue is justice. Am J Public Health. (2011) 101:S149–55. doi: 10.2105/AJPH.2010.300062 21551385 PMC3222512

[2] Guagliardo MF. Spatial accessibility of primary care: concepts, methods and challenges. Int J Health Geogr. (2004) 3:3. doi: 10.1186/1476-072X-3-3 14987337 PMC394340

[3] Luo W Wang F. Measures of spatial accessibility to health care in a GIS environment: synthesis and a case study in the Chicago region. Environ Plan B Plan Design. (2003) 30:865–84. doi: 10.1068/b29120 34188345 PMC8238135

[4] Ye P Ye Z Xia J Zhong L Zhang M Lv L . National-scale 1-km maps of hospital travel time and hospital accessibility in China. Sci Data. (2024) 11:1130. doi: 10.1038/s41597-024-03981-y 39406783 PMC11480330

[5] Hart JT. The inverse care law. Lancet. (1971) 297:405–12. doi: 10.1016/S0140-6736(71)92410-X 4100731

[6] Kruk ME Myers M Varpilah ST Dahn BT. What is a resilient health system? Lessons from Ebola. Lancet. (2015) 385:1910–2. doi: 10.1016/S0140-6736(15)60755-3 25987159

[7] Biddle L Wahedi K Bozorgmehr K. Health system resilience: a literature review of empirical research. Health Policy Plan. (2020) 35:1084–109. doi: 10.1093/heapol/czaa032 32529253 PMC7553761

[8] World Health Organization. People's Republic of China Health System Review. Health Systems in Transition. Geneva: World Health Organization (2015).

[9] Xu J Mills A. 10 years of China's comprehensive health reform: a systems perspective. Health Policy Plan. (2019) 34:403–6. doi: 10.1093/heapol/czz026 31257418

[10] Brixi H Mu Y Targa B Hipgrave D. Engaging sub-national governments in addressing health equities: challenges and opportunities in China's health system reform. Health Policy Plan. (2013) 28:809–24. doi: 10.1093/heapol/czs120 23221008

[11] Wei Z Bai J. Measuring spatial accessibility of residents' medical treatment under hierarchical diagnosis and treatment system: multi-scenario simulation in China. PLoS ONE. (2023) 18:e0282713. doi: 10.1371/journal.pone.0282713 37036836 PMC10085051

[12] Tao Z Cheng Y Liu J. Hierarchical two-step floating catchment area (2SFCA) method: Measuring the spatial accessibility to hierarchical healthcare facilities in Shenzhen, China. Int J Equity Health. (2020) 19:164. doi: 10.1186/s12939-020-01280-7 32957992 PMC7507269

[13] Cai C Hone T Millett C. The heterogeneous effects of China's hierarchical medical system reforms on health service utilisation and health outcomes among elderly populations: a longitudinal quasi-experimental study. Lancet. (2023) 402:S30. doi: 10.1016/S0140-6736(23)02141-4 37997071

[14] Jia P Wang Y Yang M Wang L Yang X Shi X . Inequalities of spatial primary healthcare accessibility in China. Soc Sci Med. (2022) 314:115458. doi: 10.1016/j.socscimed.2022.115458 36279792

[15] Pan J Shallcross D. Geographic distribution of hospital beds throughout China: a county-level econometric analysis. Int J Equity Health. (2016) 15:179. doi: 10.1186/s12939-016-0467-9 27821181 PMC5100192

[16] Wang Z Dong L Xing X Y . Disparity in hospital beds' allocation at the county level in China: an analysis based on a Health Resource Density Index (HRDI) model. BMC Health Serv Res. (2023) 23:1293. doi: 10.1186/s12913-023-10266-4 37996897 PMC10668462

[17] Song C Fang L Xie M Tang Z Zhang Y Tian F . Revealing spatiotemporal inequalities, hotspots, and determinants in healthcare resource distribution: insights from hospital beds panel data in 2308 Chinese counties. BMC Public Health. (2024) 24:423. doi: 10.1186/s12889-024-17950-y 38336709 PMC11218403

[18] Li H Yuan B Song S Meng Q Kawachi I. Trends in resource allocation for primary health care in rural China: concentration curve and decomposition analysis. Int J Health Plann Manage. (2024) 39:1551–61. doi: 10.1002/hpm.3828 39054616

[19] Song S Yuan B Zhang L Cheng G Zhu W Hou Z . Increased inequalities in health resource and access to health care in rural China. Int J Environ Res Public Health. (2019) 16:49. doi: 10.3390/ijerph16010049 30585243 PMC6338945

[20] Uchimura H. Jütting JP. Fiscal decentralization, Chinese style: good for health outcomes? World Dev. (2009) 37:1926–34. doi: 10.1016/j.worlddev.2009.06.007

[21] Jia J Guo Q Zhang J. Fiscal decentralization and local expenditure policy in China. China Econ Rev. (2014) 28:107–22. doi: 10.1016/j.chieco.2014.01.002

[22] Zhang X Kanbur R. Spatial inequality in education and health care in China. China Econ Rev. (2005) 16:189–204. doi: 10.1016/j.chieco.2005.02.002

[23] Chen C Zheng M Zhu J. How does fiscal decentralization affect health outcomes? evidence from China. Risk Manage Healthc Policy. (2023) 16:1893–903. doi: 10.2147/RMHP.S412547 37736599 PMC10511016

[24] Xu W Lin J. Fiscal decentralization, public health expenditure and public health–evidence from China. Front Public Health. (2022) 10:773728. doi: 10.3389/fpubh.2022.773728 35664120 PMC9157548

[25] Abimbola S Baatiema L Bigdeli M. The impacts of decentralization on health system equity, efficiency and resilience: a realist synthesis of the evidence. Health Policy Plan. (2019) 34:605–17. doi: 10.1093/heapol/czz055 31378811 PMC6794566

[26] Sapkota S Dhakal A Rushton S van Teijlingen E Marahatta SB Balen J . The impact of decentralisation on health systems: a systematic review of reviews. BMJ Global Health. (2023) 8:e013317. doi: 10.1136/bmjgh-2023-013317 38135299 PMC10749071

[27] Grömping U. Relative importance for linear regression in R: the package relaimpo. J Stat Softw. (2007) 17:1–27. doi: 10.18637/jss.v017.i01

[28] Moran PAP. Notes on continuous stochastic phenomena. Biometrika. (1950) 37:17–23. doi: 10.1093/biomet/37.1-2.17 15420245

[29] Anselin L. Local indicators of spatial association—LISA. Geogr Anal. (1995) 27:93–115. doi: 10.1111/j.1538-4632.1995.tb00338.x

[30] Zhang X Zhao L Cui Z Wang Y. Study on equity and efficiency of health resources and services based on key indicators in China. PLoS ONE. (2015) 10:e0144809. doi: 10.1371/journal.pone.0144809 26679187 PMC4683010

[31] Su W Du L Fan Y Wang P. Equity and efficiency of public hospitals' health resource allocation in Guangdong Province, China. Int J Equity Health. (2022) 21:138. doi: 10.1186/s12939-022-01741-1 36138478 PMC9493174

[32] Dong E Liu S Chen M Wang H Chen LW Xu T . Differences in regional distribution and inequality in health-resource allocation at hospital and primary health centre levels: a longitudinal study in Shanghai, China. BMJ Open. (2020) 10:e035635. doi: 10.1136/bmjopen-2019-035635 32690509 PMC7371131

[33] Yang L Sun L Wen L Zhang H Li C Hanson K . Financing strategies to improve essential public health equalization and its effects in China. Int J Equity Health. (2016) 15:194. doi: 10.1186/s12939-016-0482-x 27905941 PMC5134004

[34] Zhang H Shi L Yang J Sun G. Efficiency and equity of bed utilization in China's health institutions: based on the rank-sum ratio method. Int J Equity Health. (2023) 22:177. doi: 10.1186/s12939-023-01986-4 37660026 PMC10474721

[35] Fang P Cao X Huang Y . How does the platform economy affect urban system: Evidence from business-to-business (B2B) e-commerce enterprises in China. Buildings. (2025) 15:1687. doi: 10.3390/buildings15101687

[36] Fang P Wang Z Huang Y . Administrative fragmentation and functional integration: quantifying urban interstice dynamics in jurong using mobile origin–destination (OD) flows. Appl Sci. (2025) 15:5675. doi: 10.3390/app15105675

[37] Sun X Xu L Adnan KMM Luo Y. Can comprehensive medical reform improve the efficiency of medical resource allocation? Evidence from China. Int J Public Health. (2023) 68:1606602. doi: 10.3389/ijph.2023.1606602 38179320 PMC10764414

[38] Lin K Yao Y Xiong Y Xiang L. The effect of an innovative payment method on inpatient volume and bed resources and their regional distribution: the case of a central province in China. Int J Equity Health. (2024) 23:159. doi: 10.1186/s12939-024-02243-y 39138482 PMC11320847

